# Formation pathways of polycyclic aromatic hydrocarbons (PAHs) in butane or butadiene flames†

**DOI:** 10.1039/d0ra08744k

**Published:** 2021-02-02

**Authors:** Tingting Zhang, Guizhi Mu, Shourong Zhang, Jialin Hou

**Affiliations:** School of Mechanical and Electrical Engineering, Shandong Agricultural University Taian 271018 P. R. China jialinhou@163.com; Department of Traffic Engineering, Shandong Transport Vocational College Taian 271000 P. R. China

## Abstract

The reaction pathways from phenyl radicals to phenanthrene (A_3_) and pyrene (A_4_) *via* C_2_H_3_ and C_4_H_4_ additions were investigated using the G3(MP2, CC) method. Rate constants of elementary reactions were calculated. The influence of additions, H-abstraction ways and reactive sites on the reaction rates were considered. These polycyclic aromatic hydrocarbon (PAH) formation pathways were used to improve the combustion chemistry model for C_4_ fuels, and the results from the improved model and the original model were compared with experimental data. H atoms are important for PAH formation owing to their influential roles in the production of aromatic radicals and stable aromatic structures. C_2_H_3_ and C_4_H_4_ addition reactions can occur at low temperature, and need less energy than C_2_H_2_ addition. The PAH formation pathways determined from G3 calculations, which were used to improve the model, were effective in promoting PAH formations in this model. Comparison of PAH formation in butane and butadiene flames showed both the C_2_H_3_ and C_4_H_4_ addition pathways included in this work can improve the formation of PAHs in butadiene and butane flames. C_4_H_4_ addition pathways in a butane flame were better for PAH formation than C_2_H_3_ addition.

## Abstract 2

The reaction pathways from phenyl radicals to phenanthrene (A_3_) and pyrene (A_4_) *via* C_2_H_3_ and C_4_H_4_ additions were investigated using the G3(MP2, CC) method.

## Graphical abstract

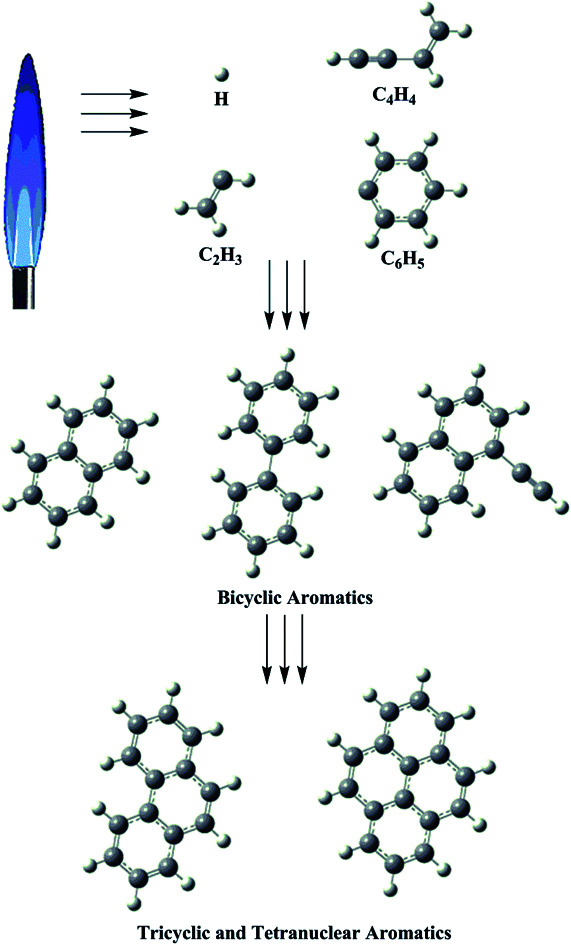

## 1. Introduction

Polycyclic aromatic hydrocarbons (PAHs), which mainly come from the incomplete combustion of fuel, are extremely harmful to humans and the environment. So far, the most commonly-used addition for PAH growth is C_2_H_2_, and the most popular PAH formation mechanism is H-abstraction-C_2_H_2_-addition (HACA).^1–5^ However, the HACA mechanism underestimates the concentrations of PAHs and soot compared with the experimental results.^6–9^ This underestimation may be explained by two main reasons. Firstly, there are many non-acetylene organic compounds (*e.g.* CH_3_, C_3_H_3_, C_3_H_4_, C_3_H_6_, C_4_H_2_, C_4_H_4_, C_4_H_6_ and C_5_H_8_) that also contribute to PAH growth in various flame configurations.^8–13^ Secondly, many PAH formation reactions in the HACA mechanism are reversible.^14^ Hence, more additions in PAH formation should be considered to perfect the PAH formation mechanism.

Many non-acetylene additions have been proved experimentally to be important for PAHs formation. C_4_H_4_ addition can effectively lead to PAHs formation.^15^ C_2_H_3_ addition is critical for the formation of PAHs and soot.^16^ The hydrogen abstraction/vinyl radical addition (HAVA) mechanism may be the best complement to the HACA mechanism, and the phenyl addition/cyclization (PAC) mechanism can promote the growth of molecular mass and ring number of PAHs without the limit of reactive sites.^10,17^ Hence, the additions of C_2_H_3_, C_4_H_4_ and C_6_H_5_ contribute greatly to PAHs formation, and more research on PAHs formation from C_2_H_3_, C_4_H_4_ and C_6_H_5_ additions is needed.

Butane and butadiene, two light hydrocarbons, are both beneficial to the environment and can relieve energy shortage. In this research, PAHs formation routes *via* C_2_H_3_, C_4_H_4_ and C_6_H_5_ additions within all reaction rates of elementary reactions in the modified Arrhenius equation were studied and coupled to a chemical kinetic model for butadiene and butanol. The effects of additions, reaction types and reaction sites were considered during PAHs formation.

## 2. Methods

All molecular structures involved in the reactions under study were optimized by using the hybrid B3LYP functional with the 6-311++G(d,p) basis set.^18–20^ Vibrational frequency was calculated at the same level to identify the optimized structures as local minimum or first-point saddle points and to provide their zero-point vibrational energy (ZPE). Intrinsic reaction coordinate calculations were implemented to ensure that the transition states connect to relevant reactants and products correctly. The final single-point energies of all species were determined using the G3(MP2, CC) method,^21,22^ which is expected to generate relative energies of various species within the accuracy of 1–2 kcal mol^−1^ (ref. 23) and is extensively used to study PAHs growth mechanisms.^21,24–28^ The intermediate species and transition structures found in this study were all closed shell singlets or open shell doubles. Molecular properties of chemical species (CS) and transition states (TS) are shown in ESI.† The G3(MP2, CC) energies are calculated as follows:

1 *E*[G3(MP2, CC)] = *E*[CCSD/6-311++(d,p)] + Δ*E*(MP2) +Δ*E*(SO) + Δ*E*(HLC) + *E*(ZPE)

where Δ*E*(MP2) = *E*[MP2/6-311G++(3df,2p)] − *E*[MP2/6-311G++(d,p)] is the basis set correction; Δ*E*(SO) is the spin–orbit correction, included for atomic species only. For molecules, higher level correction (HLC) is Δ*E*(HLC) = −*An*_b_ − *B*(*n*_a_ − *n*_b_) with *A* = 9.279 mHartrees and *B* = 4.471 mHartrees, and for atoms, is Δ*E*(HLC) = −*Cn*_b_ − *D*(*n*_a_ − *n*_b_) with *C* = 9.345 mHartrees and *D* = 2.021 mHartrees, where *n*_a_ and *n*_b_ are the numbers of *a* and *b* valence electrons, respectively. All calculations were performed on Gaussian 09.^29^

Based on the calculated potential energy surface (PES) and molecular characteristics, Rice–Ramsperger–Kassel–Marcus (RRKM) and transitional state theory (TST) were used to determine reaction rates by ChemRate program.^30^ Noticeably, none of the rate constants were arbitrarily changed or intuitively estimated to match the computed and the experimental results. The calculated PAHs formation pathways were used to update Hansen's mechanism^31^ which consists of 216 species connected *via* 1028 reactions. Then the updated mechanism was used to simulate the premixed 1,3-butadiene or butane flame and to discuss the roles of our pathways in PAHs formation under 1,3-butadiene or butane flame.

## 3. Results and discussion

### 3.1 PAHs formation pathways

**Fig. 1:**
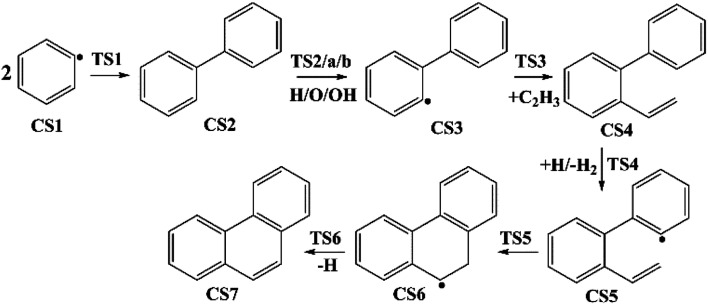
Phenanthrene formation pathways from phenyl radical *via* C_6_H_5_ and C_2_H_3_ additions.

**Fig. 2:**
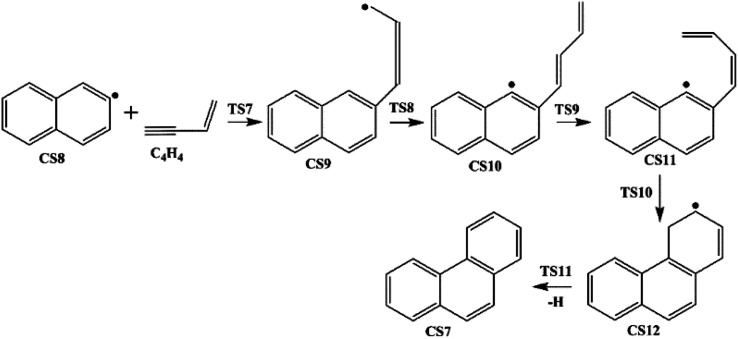
Phenanthrene formation pathways from naphthyl radical *via* C_4_H_4_ addition.

**Fig. 3:**
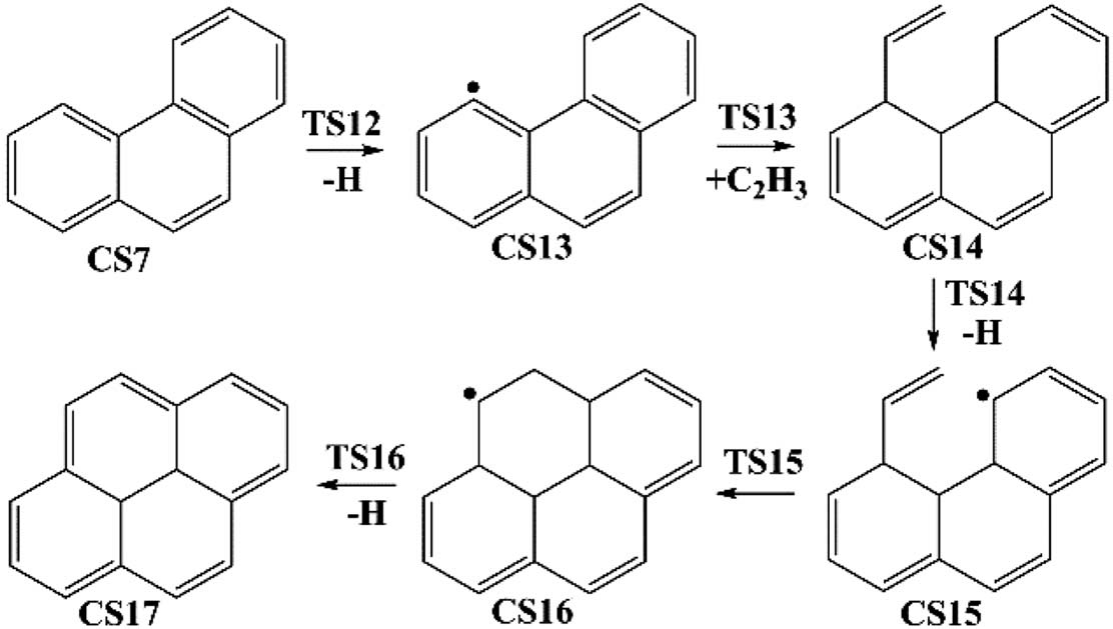
Pyrene formation pathways from phenanthryl radical *via* C_2_H_3_ addition.

**Fig. 4:**
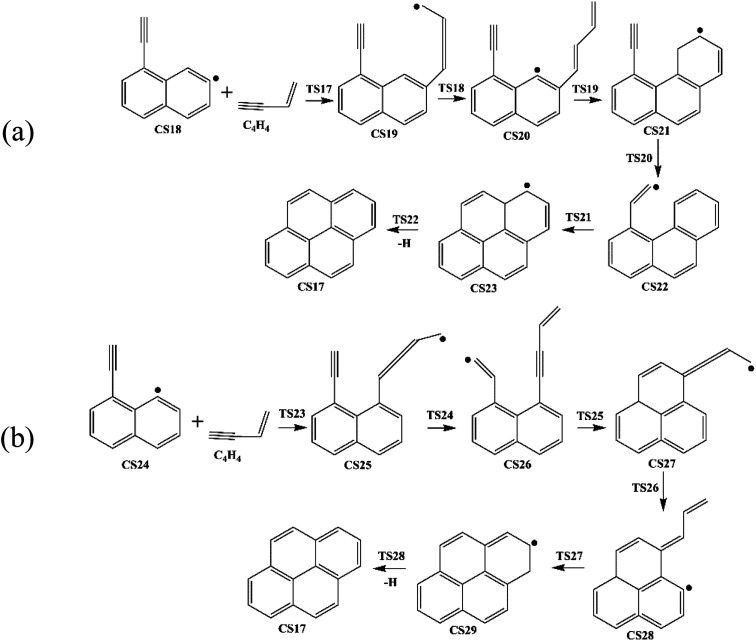
Pyrene formation pathways *via* C_4_H_4_ addition onto different sites of 1-ethynylnaphthyl radical.

Four PAHs formation pathways were studied (Fig. 1–4): (i) formation of phenanthrene *via* once addition of phenyl radical (C_6_H_5_) and twice additions of C_2_H_3_ onto phenyl radical; (ii) formation of phenanthrene *via* once addition of C_4_H_4_ onto naphthyl radical; (iii) formation of pyrene *via* once addition of C_2_H_3_ onto phenanthryl radical; (iv) formation of pyrene *via* once addition of C_4_H_4_ onto 1-ethynylnaphthyl radical. Comparisons between H abstraction reactions of biphenyl *via* H atom, O atom or OH radical at same reactive site were studied. The effects of reactive sites to hydrogen abstraction, carbon addition, ring closure, and hydrogen atom loss reactions were investigated. The barrier heights and reaction energies for all steps are collected in Table S1.† Meanwhile, to understand the thermodynamics of the studied reactions better, the enthalpies and Gibbs free energies are illustrated in Tables S2 and S3.† The molecular geometries, vibrational frequencies, moments of inertia, and rotational constants of the chemical species (CS) and transition states (TS) involved in all routes are shown in Table S5.†

#### 3.1.1 Phenanthrene formation pathway *via* C_2_H_3_ additions

As shown in Fig. 1, phenanthrene was formed through H-abstractions, C-additions and ring formation reactions, and the barrier heights and reaction energies were determined in Table 1. The initial association of two C_6_H_5_ additions results in the formation of CS2, biphenyl. This thermodynamically and kinetically favorable process is highly exothermic (119.7 kcal mol^−1^) with a barrier of 14.3 kcal mol^−1^, leading to a quick increase of ring number. In Fig. 6, C_2_H_2_, and especially C_6_H_5_ and C_2_H_3_ addition reactions are all exothermic. Besides, C_2_H_3_ addition reaction occurs more easily than C_2_H_2_ or C_6_H_5_ addition reaction.

**Table 1:** Barrier heights and reaction energies for steps involved in phenanthrene formation pathways from phenyl radical *via* C_6_H_5_ and C_2_H_3_ additions computed at the G3(MP2, CC) level

No.	Reaction	Barriers/kcal mol^−1^	Reaction heats/kcal mol^−1^
**Carbon addition**			
R1	2CS1 = CS2	14.3	−119.7
R3	CS3 + C_2_H_3_ = CS4	3.8	−116.2

**Hydrogen abstraction**			
Ra	CS2 + O = CS3 + OH	292.3	16.7
Rb	CS2 + OH = CS3 + H_2_O	436.5	0.5
Rc	CS2 + C_2_H_3_ = CS3 + C_2_H_4_	18.1	7.4
Rd	CS2 + C_6_H_5_ = CS3 + C_6_H_6_	14.4	3.0
R2	CS2 + H = CS3 + H_2_	22.4	13.1
R4	CS4 + H = CS5 + H_2_	23.6	13.0

**Ring formation**			
R5	CS5 = CS6	7.3	−39.7

**Hydrogen loss and (or) disproportionation**			
R6	CS6 = CS7 + H	27.9	16.5

**Fig. 5:**
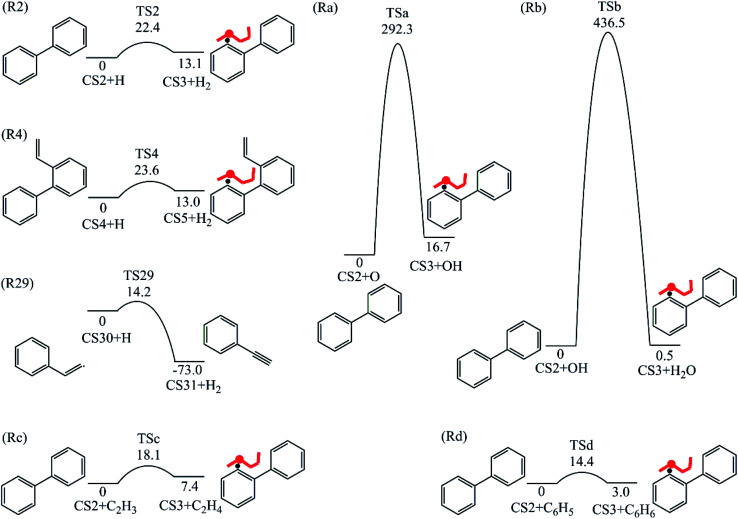
H-abstractions *via* the assistance of H, O, OH, C_2_H_3_ or C_6_H_5_ radicals.

H-abstractions may produce singlet PAH intermediates such as CS31 and CS7, or radicals such as CS3 and CS5.^32^ Most of the former H-abstractions are exothermic, and most of the later H-abstractions are endothermic, providing precondition for the possible carbon additions or cyclization reactions. To explore the easier formation of larger PAHs molecules, we calculated and compared five radical site formation processes *via* H, O, OH, C_2_H_3_ and C_6_H_5_ respectively to provide a prime radical formation process. The barriers and reaction heats were shown in Table 1 and all H-abstraction reactions with assistance of H, O, OH, C_2_H_3_ and C_6_H_5_ in this pathway together with H-abstraction reaction R29 from literature^32^ were compared in Fig. 5. Comparison of R2, Ra, Rb, Rc and Rd showed the barriers of the H-abstraction reactions *via* O and OH radicals were much higher than those *via* H, C_2_H_3_ and C_6_H_5_ radicals. This means that, depending on the concentrations of H, C_2_H_3_ and C_6_H_5_ radicals in combustion environments, H, C_2_H_3_ and C_6_H_5_ radicals can much more easily initiate H-abstractions than O and OH radicals. However, the concentration H radicals in combustion environments is generally higher than those of C_2_H_3_ and C_6_H_5_ radicals.^8–10^ Thus, H radical is much easier to initiate H-abstractions than O, OH, C_2_H_3_ and C_6_H_5_ radicals. Little difference between H-abstractions *via* TS2 and TS4 was found in barriers or reaction heats, which is mainly because of the same reaction types and reactive sites between armchair and free edges.^33^ CS6 was formed *via* a cyclization reaction after H-abstraction, and formed CS7, phenanthrene, by emitting a H atom.

**Fig. 6:**
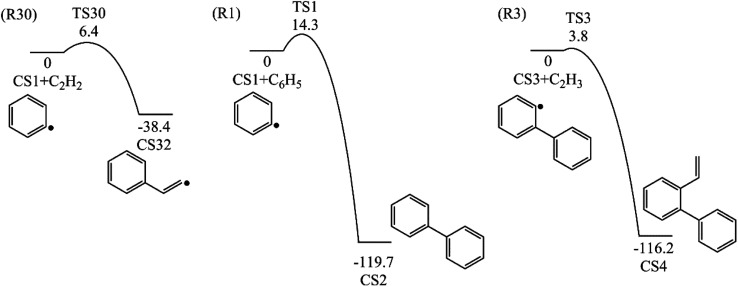
C_2_H_2_, C_6_H_5_ and C_2_H_3_ addition reactions.

#### 3.1.2 Phenanthrene formation pathway *via* C_4_H_4_ addition

**Fig. 7:**
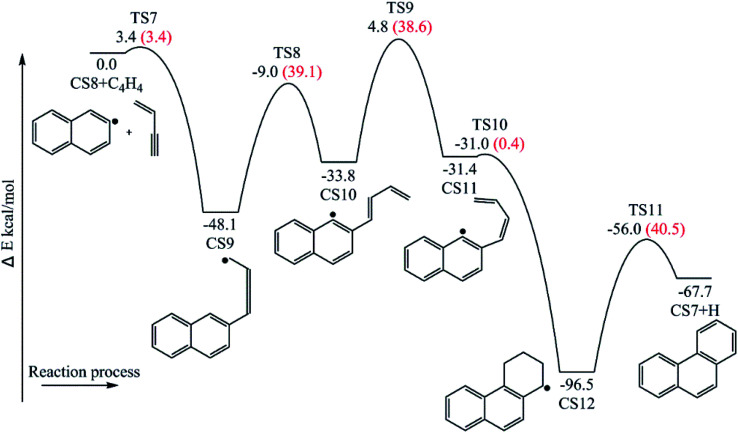
Phenanthrene formation initiated by C_4_H_4_ addition onto naphthyl radical.

This pathway describes the phenanthrene formation process initiated by C_4_H_4_ addition onto naphthyl radical. The energies of the intermediate species and the transition states are relative to the total energy of CS8 and C_4_H_4_ (Fig. 7). The addition of C_4_H_4_ leads to CS9 with a barrier of 3.4 kcal mol^−1^, which is lower than that of C_2_H_2_ addition, RI. After that, the reaction CS9 → CS10 is internal hydrogen abstraction *via* migration of a hydrogen atom from the aromatic ring to the second carbon of the C4 fragment, producing a butadiene chain with a barrier of 39.1 kcal mol^−1^. Then to prepare for the cyclization, CS10 undergoes *cis*–*trans* isomerization, forming CS11 with a barrier of 38.6 kcal mol^−1^. Due to its structural characteristics, the CS11 adduct can be cyclized, producing the phenanthrene precursor CS12, which yields phenanthrene after hydrogen atom elimination. The barrier of the cyclization step CS11 → CS12 is only 0.4 kcal mol^−1^, which can be ignored easily. Also, the reaction is highly exothermic by 65.1 kcal mol^−1^. The hydrogen atom elimination step CS12 → CS7 + H overcomes a barrier of 40.5 kcal mol^−1^, and is the rate determining step of this pathway.

#### 3.1.3 Pyrene formation pathway *via* C_2_H_3_ additions

The reaction process from phenanthrene to pyrene *via* C_2_H_3_ addition and the energy of this process were shown in Fig. 3 and Table 2. The C_2_H_3_ addition reaction is followed by activation of phenanthrene *via* H-abstraction, forming a radical structure, CS13. After that, the C_2_H_3_ addition onto CS13 is highly exothermic with a barrier of only 5.4 kcal mol^−1^. Then CS14 undergoes H-abstraction and cyclization, producing CS16, with barriers of 31.0 and 5.3 kcal mol^−1^, respectively. At last, CS16 emits a H atom to produce CS17, pyrene. This reaction is a rate-determining step of this pathway, overcoming a barrier of 38.2 kcal mol^−1^.

**Table 2:** Barrier heights and reaction energies for steps involved in pyrene formation pathway from phenanthrene *via* C_2_H_3_ addition computed at the G3(MP2, CC) level

No.	Reaction	Barrier/kcal mol^−1^	Reaction heat/kcal mol^−1^
R13	CS13 + C_2_H_3_ = CS14	5.4	−113.0
R14	CS14 + H = CS15 + H_2_	31.0	11.9
R15	CS15 = CS16	5.3	−53.2
R16	CS16 = CS17 + H	38.2	25.0

**Fig. 8:**
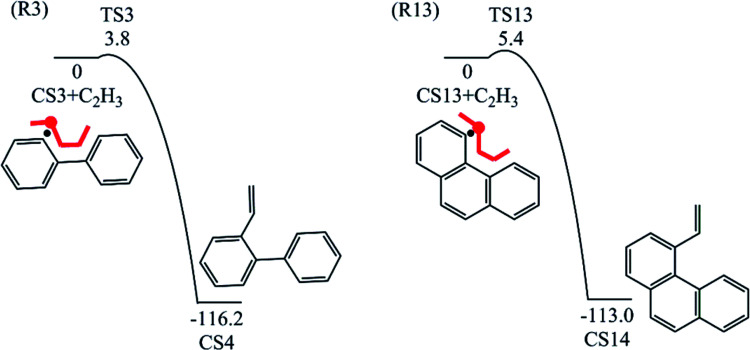
C_2_H_3_ addition reactions onto CS3 and CS13.

Fig. 8 compares two C_2_H_3_ additions onto CS3 and CS13 separately. Little difference is found in the barrier heights or reaction energy between these two addition steps. This may be because the reactive sites on CS3 and CS13 are similar between “armchair” and “free edge” as shown by red lines in Fig. 8.

**Fig. 9:**
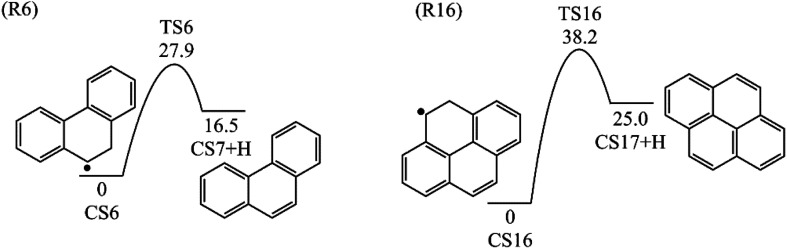
H atom loss reactions.

Fig. 9 shows two hydrogen atom loss reactions that produce phenanthrene and pyrene, respectively. Compared with H-abstractions *via* the assistance of H atom, these two H atom loss reactions without the assistance of H atom need to overcome higher barriers and are both endothermic. Thus, the assistance of H atom is beneficial for H loss.

**Fig. 10:**
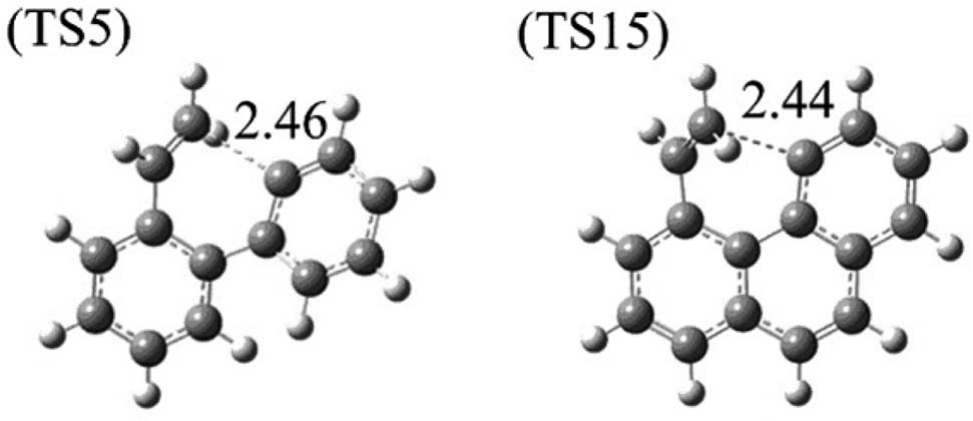
Structures of transition states of R5 and R15.

Furthermore, both reaction CS15 → CS16 and reaction CS5 → CS6 are ring formation processes that occur at the edge of vinyl group and the carbon of aromatic ring between “armchair” and “free edge” (Fig. 1, 3 and 10). These two reactions are both exothermic with low barriers.

#### 3.1.4 Pyrene formation pathways *via* C_4_H_4_ addition onto CS18

**Fig. 11:**
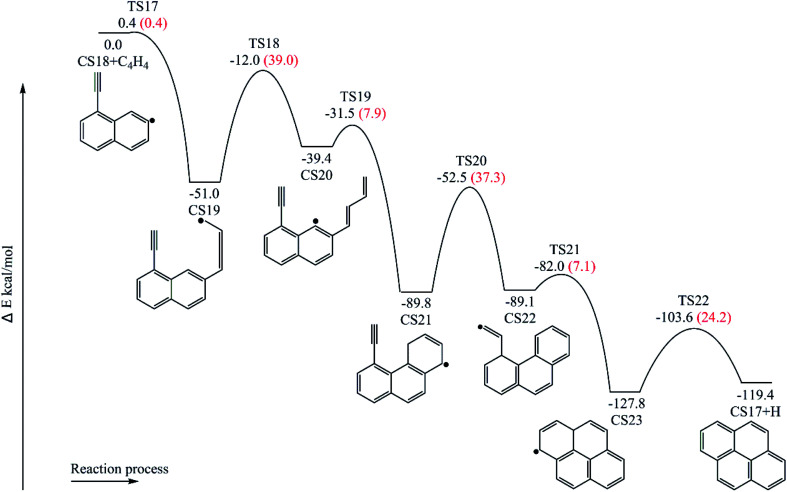
Pyrene formation pathways *via* C_4_H_4_ addition onto CS18.

Fig. 11 provides the energy diagram for pyrene formation pathway initiated from the C_4_H_4_ addition onto CS18. The energies of the intermediate species and the transition states are relative to the total energy of CS18 and C_4_H_4_. The C_4_H_4_ addition onto the radical site of CS18 to form CS19 requires a barrier of 0.4 kcal mol^−1^, which is 3.0 kcal mol^−1^ lower than the C_4_H_4_ addition step above, CS8 + C_4_H_4_ → CS9. This may be because of the existence of the acetylene group in CS18. After that, the reaction CS19 → CS20 is internal hydrogen abstraction *via* migration of a hydrogen atom, producing a butadiene chain with a barrier of 39.0 kcal mol^−1^. The energetics of this reaction is rather similar to that observed for another internal hydrogen migration step CS9 → CS10. The barrier heights range within 39.0–39.1 kcal mol^−1^, and reaction exothermicities are about 11.6–14.3 kcal mol^−1^. After that, cyclization occurs at the end of the butadiene group and the radical site of aromatic ring, CS20 → CS21, which requires an activation energy of 7.9 kcal mol^−1^, forming a six-membered ring. Later, another cyclization process takes place at the vinyl radical group and the newly-formed ring, forming the forth ring structure, CS23, with a barrier of 7.1 kcal mol^−1^. Finally, adduct CS23 eliminates an “extra” hydrogen atom to produce CS17, pyrene.

**Fig. 12:**
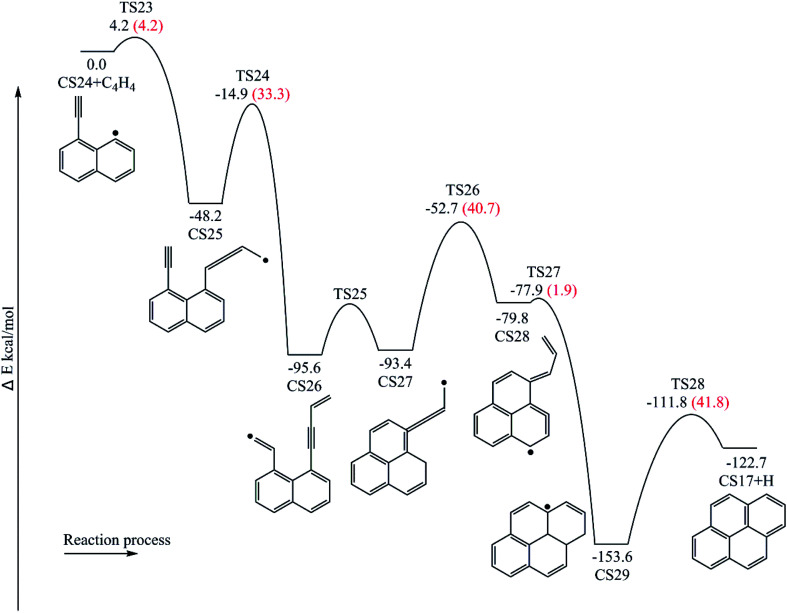
Pyrene formation pathway *via* C_4_H_4_ addition onto CS24.

Fig. 12 provides the energy diagram for pyrene formation pathway initiated from the C_4_H_4_ addition onto CS24. The energies of the intermediate species and the transition states are relative to the total energy of CS18 and C_4_H_4_. The C_4_H_4_ addition onto the radical site of CS24 to produce CS25 requires a barrier of 4.2 kcal mol^−1^, which is higher than C_4_H_4_ addition steps *via* TS7 and TS17. This may be because the reactive site for C_4_H_4_ addition is near the shared carbons of two aromatic rings. The exothermicity of this addition reaction is 48.2 kcal mol^−1^, which differs by only 0.1 and 2.8 kcal mol^−1^ with the other two C_4_H_4_ addition steps *via* TS7 and TS17, respectively, but is significantly lower than the reaction energies for C_6_H_5_ and C_2_H_3_ addition reactions, R1, R3 and R13. Combined with Fig. 6 and 8, in the addition reactions, the same added species may contribute equally to similar reaction kinetics, just like C_2_H_3_ and C_4_H_4_. After the addition of C_4_H_4_, the internal hydrogen migration from C4 group to acetylene group adjusts the structure of CS25 to adduct CS26, which yields CS27 within three aromatic rings through cyclization *via* TS25. At this point, another internal hydrogen migration *via* TS26 has a large thermodynamic driving force for the subsequent formation of PAHs, CS29. At last, pyrene is formed through the elimination of an “extra” hydrogen atom from adduct CS29. In this pathway, the structure of TS25, which is the transition state for CS26 → CS27, was not found. Thus, the highest barrier of this pathway is 41.8 kcal mol^−1^ so far, and the rate-deciding step is the dehydrogenation, CS29 → CS17 + H.

### 3.2 Elementary reaction rate constants

**Fig. 13:**
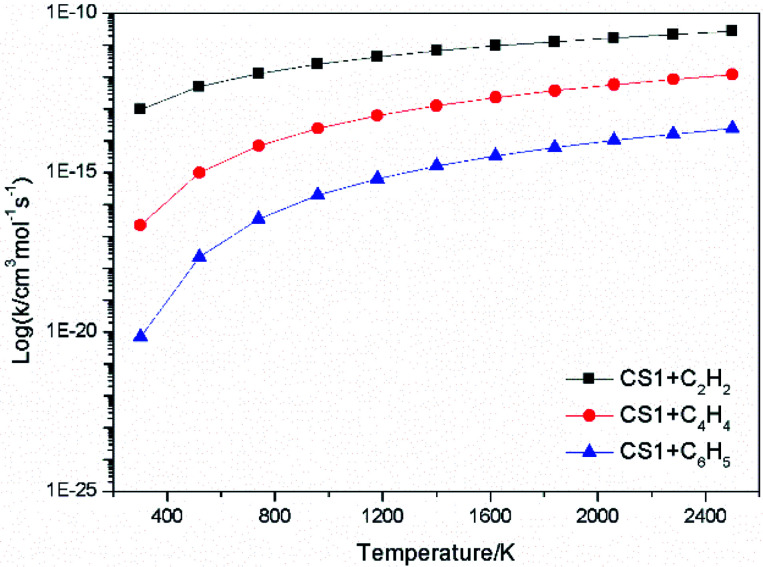
Elementary reaction rate constants for C_2_H_2_, C_4_H_4_ and C_6_H_5_ additions.

**Fig. 14:**
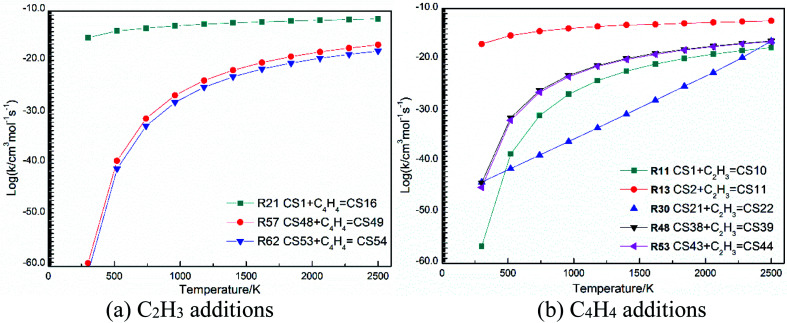
Elementary reaction rate constants for C_2_H_3_ and C_4_H_4_ additions along temperature.

**Fig. 15:**
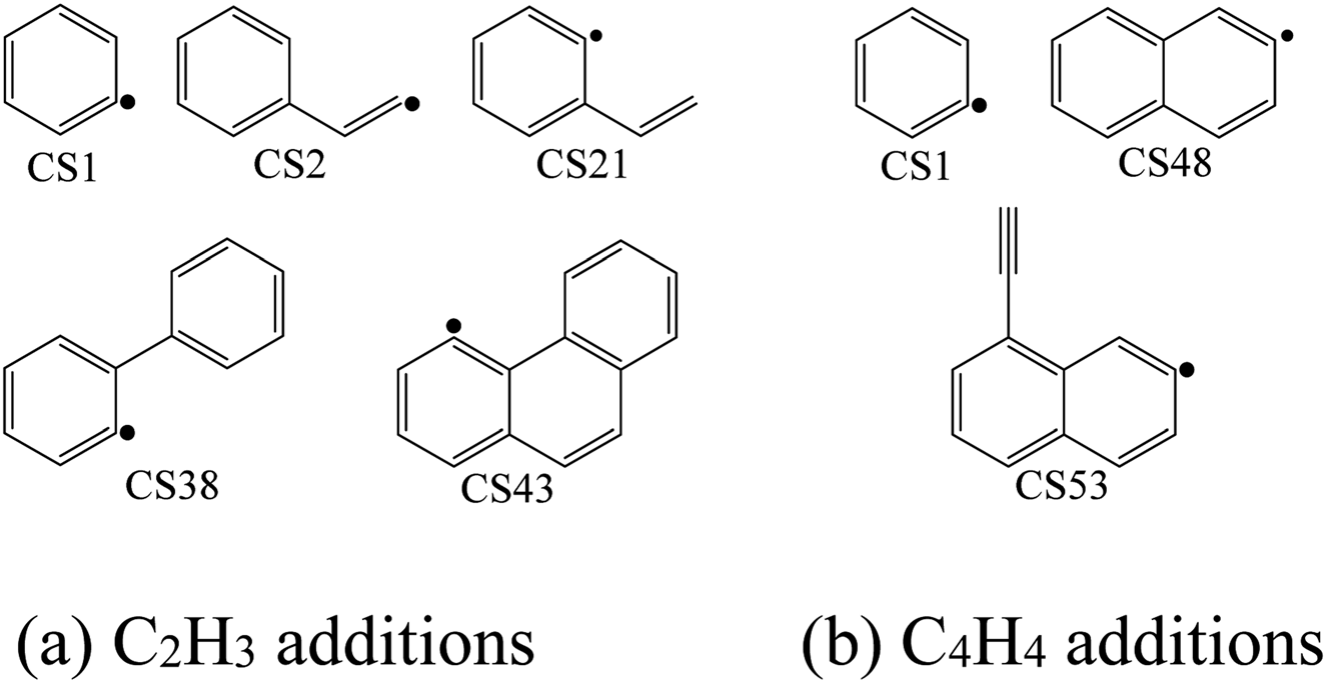
Aromatic structures for C_2_H_3_ and C_4_H_4_ additions.

The high-pressure limit rate constants of the elementary reactions involved above are listed in Table 3 ranging from 300 to 2500 K. Fig. 13 and 14 demonstrate the rate constants for C_2_H_2_, C_4_H_4_ and C_6_H_5_ additions, and for C_2_H_3_ and C_4_H_4_ additions along temperature respectively. Fig. 15 shows the aromatic structures for C_2_H_3_ and C_4_H_4_ additions. Results imply that reactions R48 and R53 almost share the same rate constants at different temperatures, which is because the reactive sites of CS38 and CS43 are both armchair sites. Moreover, for C_4_H_4_ additions, the rate constant added onto CS1 is the fastest, followed by CS48, and that of CS53 is the last. All reactive sites on CS1, CS48 and CS53 are free edges with no adjacent groups, and the molecular mass of CS1 is the lightest, followed by CS48, and that of CS53 is the heaviest. Thus, we believe C_4_H_4_ can be more easily added onto aromatic structures with light mass.

**Table 3:** Elementary reaction rate constants in the modified Arrhenius equation form of *AT*^*n*^ exp(−*E*/*RT*) with the units of cal, K, mol, cm and s, evaluated at 300–2500 K

No.	Reaction	*A*	*n*	*E*
**Phenanthrene formation pathway *via* C**_**2**_ **H**_**3**_ **addition**				
R1	2CS1 → CS2	5.00 × 10^−14^	−0.3	8.2
R2	CS2 + H → CS3 + H_2_	4.00 × 10^−16^	1.8	23
R3	CS3 + C_2_H_3_ → CS4	5.00 × 10^−11^	−1.1	42.4
R4	CS4 + H → CS5 + H_2_	4.00 × 10^−16^	1.8	23.8
R5	CS5 → CS6	4.00 × 10^12^	0.4	10.3
R6	CS6 → CS7 + H	3.20 × 10^10^	1	42.3

**Phenanthrene formation pathway *via* C**_**4**_ **H**_**4**_ **addition**				
R7	CS8 + C_4_H_4_ → CS9	3.40 × 10^16^	−9.6	89.9
R8	CS9 → CS10	8.40 × 10^62^	−15.2	57.2
R9	CS10 → CS11	3.90 × 10^30^	−5.9	28.9
R10	CS11 → CS12	6.10 × 10^12^	0.1	1.5
R11	CS12 → CS7 + H	2.00 × 10^11^	0.8	29.5

**Pyrene formation pathway *via* C**_**2**_ **H**_**3**_ **addition**				
R12	CS7 + H → CS13 + H_2_	—	—	—
R13	CS13 + C_2_H_3_ → CS14	5.90 × 10^10^	−1.4	44.6
R14	CS14 + H → CS15 + H_2_	2.50 × 10^−16^	1.8	27.5
R15	CS15 → CS16	2.50 × 10^12^	0.1	5.8
R16	CS16 → CS17 + H	5.00 × 10^10^	1.0	43.6

**Pyrene formation pathways *via* C**_**4**_ **H**_**4**_ **addition**				
R17	CS18 + C_4_H_4_ → CS19	4.30 × 10^39^	−10.4	90.4
R18	CS19 → CS20	4.00 × 10^11^	0.4	42.2
R19	CS20 → CS21	5.80 × 10^11^	0.1	8
R20	CS21 → CS22	8.70 × 10^10^	0.5	38.9
R21	CS22 → CS23	1.60 × 10^12^	0.1	7.9
R22	CS23 → CS17 + H	1.80 × 10^11^	0.8	29.5
R23	CS24 + C_4_H_4_ → CS25	8.60 × 10^49^	−13.9	94.7
R24	CS25 → CS26	1.20 × 10^32^	0.5	36.6
R25	CS26 → CS27	—	—	—
R26	CS27 → CS28	3.30 × 10^32^	0.4	44.1
R27	CS28 → CS29	3.10 × 10^32^	0.6	2.3
R28	CS29 → CS17 + H	8.90 × 10^30^	0.6	47.5

### 3.3 Kinetic modeling

**Fig. 16:**
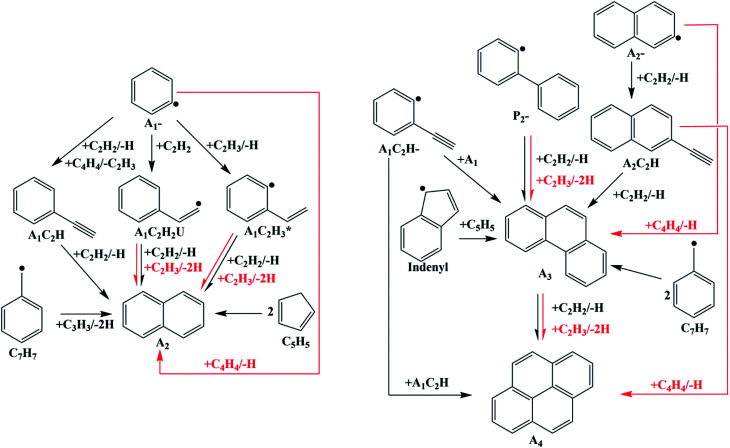
PAHs formation process in the improved mechanism (black lines: from Hansen's mechanism; red lines: from quantum chemical calculations reported before^32^ and in this study).

The mechanism investigated here was improved from Hansen's mechanism^31^ by the quantum chemical calculations reported before^32^ and in this work. The improvements of the new mechanism are shown in Fig. 16. All computations for laminar flames were performed with the code PREMIX from CHEMKIN II.^34^ Thermodynamic and transport data for the species involved in the mechanism were taken from Hansen's mechanism^31^ or evaluated by applying group additives rules.

The improved mechanism was verified in premixed butane and butadiene flames separately. Species concentrations determined from these flames were compared with the experimental results and the simulation results from Hansen's mechanism. Thereby, the influence of new PAHs formation routes on predicting PAHs formation was analyzed.

#### 3.3.1 Species concentration in premixed 1,3-butadiene flame

**Fig. 17:**
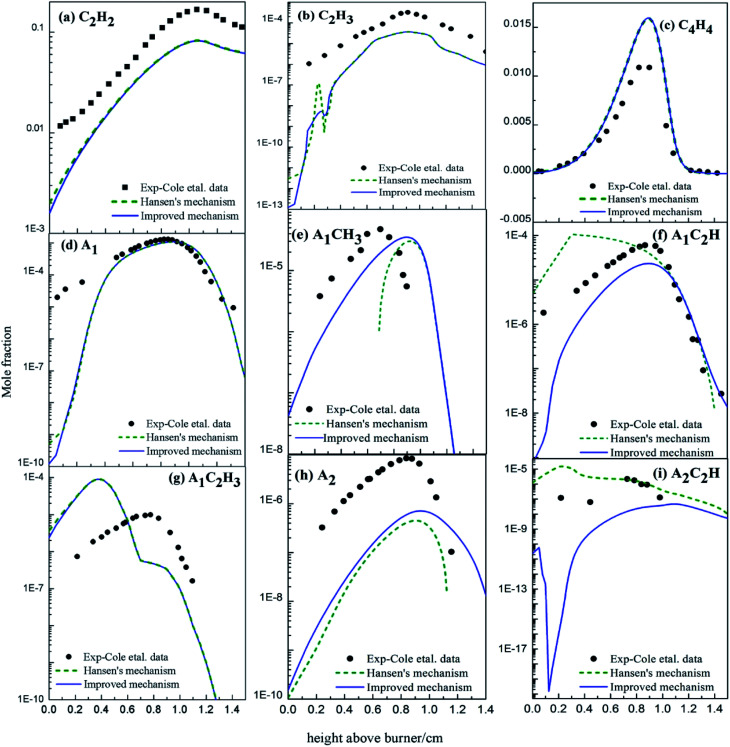
Comparison of calculated and experimental data^35^ of mole fraction profiles for major products in butadiene flames (black dots: from experiments;^35^ dashed lines: from Hansen's mechanism;^31^ solid lines: from the improved mechanism).

With the improved chemical mechanism, we simulated the variation in concentrations of the main intermediates and PAHs in the premixed 1,3-butadiene flame. All modelling parameters were cited from experiments,^35^ and all results were shown in Fig. 17 compared with experiments^35^ and simulation of Hansen's mechanism.^31^

The mole concentrations of C_2_H_2_, C_2_H_3_, C_4_H_4_, A_1_ and A_1_C_2_H_3_ decrease at low temperature (Fig. 17). Among them, the consumption of C_2_H_3_, C_4_H_4_, A_1_ and A_1_C_2_H_3_ may be because of the newly-added PAHs formation routes, and the C_2_H_2_ concentration decreases probably because of the transition of C_2_H_2_ into C_2_H_3_ and C_4_H_4_ for chemical equilibrium. Specifically, the added PAHs formation routes do contribute to PAHs formation, especially at low temperature.

The mole concentrations of A_1_CH_3_ and A_2_ increase, but those of A_1_C_2_H and A_2_C_2_H decrease compared with the results of Hansen's mechanism. A_2_ forms from HACA routes, conjugation of A_1_CH_2_ radical and C_3_H_3_ radical, or conjugation of two cyclopentadienyl radical in Hansen's mechanism (Fig. 16). A_2_ formation routes *via* C_2_H_3_ and C_4_H_4_ additions are newly-added in this study. Hence, the increase of the A_2_ mole concentration compared to the results of Hansen's mechanism is mainly due to the new added routes. As a consequence of chemical equilibrium, the mole concentration of A_1_CH_3_ also increases. Since A_1_C_2_H and A_2_C_2_H are both reactants in the new added PAHs formation routes, their mole concentrations decrease compared with the results of Hansen's mechanism.

#### 3.3.2 Species concentration in premixed butane flame

**Fig. 18:**
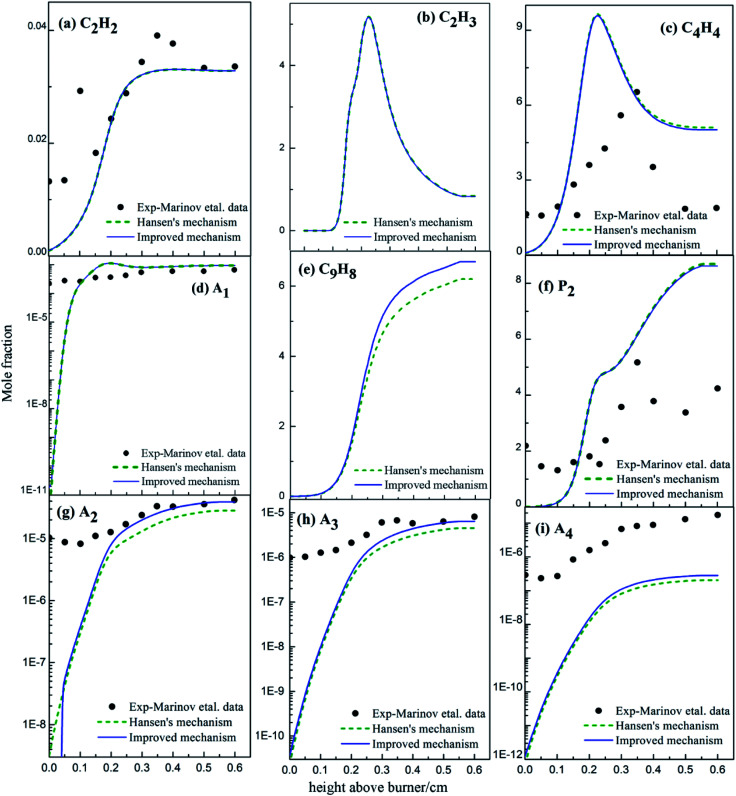
Comparison of calculated and experimental data^36^ of mole fraction profiles for major products in butadiene flames (black dots: from experiments;^36^ dashed lines: from Hansen's mechanism;^31^ solid lines: from the improved mechanism).

The variations in the concentrations of main intermediates and PAHs in the premixed butane flame were simulated according to experiments,^36^ Hansen's mechanism^31^ and the improved mechanism in this study. All results were shown in Fig. 18.

The C_2_H_3_ mole concentration from the improved mechanism agrees well with the data from Hansen's mechanism (Fig. 18). Namely, the newly-added PAHs formation routes *via* C_2_H_3_ addition were not brought into full play in butane flame. Compared with the modelling results from Hansen's mechanism, the mole concentration of C_4_H_4_ from the improved mechanism decreases, and those of A_2_, A_3_ and A_4_ from the improved mechanism increase. However, at low temperature, the concentration of A_2_ decreases obviously compared with Hansen's results for the newly-added A_3_ formation routes from A_2_. Thus, the newly-added PAHs formation routes *via* C_4_H_4_ addition play key roles in butane flame.

The mole concentration of C_9_H_8_ from the improved mechanism increases compared with results from Hansen's mechanism, because the newly-added A_3_ formation routes improve the formation of A_3_, and a part of A_3_ forms indenyl and cyclopentadienyl due to the chemical equilibrium. Hence, the newly-added PAHs formation routes, especially the C_4_H_4_ addition routes, contribute in butane flame.

Comprehensive comparison about the added PAHs formation routes and the modelling results of 1,3-butadiene and butane flame shows that both C_2_H_3_ and C_4_H_4_ addition routes benefit PAHs formation in 1,3-butadiene flame, but C_4_H_4_ addition routes benefit PAHs formations more than C_2_H_3_ additions in butane flame. This is mainly because of the geometries of 1,3-butadiene and butane.

## 4. Conclusions

Detailed A_3_ and A_4_ formation routes *via* C_2_H_3_ and C_4_H_4_ addition reactions onto aromatic radicals were investigated with the G3(MP2, CC) method. The influences of reaction sites, reaction types and additions to PAHs formation rates were discussed. The PAHs formation routes gained in this study and reported before were used to improve an existing mechanism. This improved mechanism was verified and compared to experimental results and the modelling results of the original mechanism. The contributions of C_2_H_3_ and C_4_H_4_ addition routes to PAHs formation in both butadiene and butane flame were studied.

(1) Compared with C_2_H_2_ addition, C_2_H_3_ and C_4_H_4_ addition reactions occurred more easily at the radicals of aromatics; C_2_H_3_, C_4_H_4_ and C_6_H_5_ additions were more irreversible, and the formed PAH geometries were more irreversible. All these addition reactions easily occurred in flame, and were verified to produce PAHs at low temperature in both butadiene and butane flame.

(2) H atoms are important for PAHs formation. On one hand, almost all H-abstractions with the assistance of H atoms need barriers about 30 kcal mol^−1^, and disproportionations need to overcome high barriers and are highly endothermic. That is, H atoms decreased the energies for radical production, and made the reactions more irreversible. On the other hand, H loss with the assistance of O atom and OH radical needs to overcome much higher barriers than that with the assistance of H atom. In other words, H atoms more easily induce H loss than O atoms and OH radicals.

(3) The C_2_H_3_ and C_4_H_4_ addition routes are both beneficial to PAHs formation in 1,3-butadiene flame, and the C_4_H_4_ addition route benefits PAHs formation in butane flame. This is mainly because C_4_H_4_ can be effectively formed from continuous dehydrogenations of 1,3-butadiene and butane. C_2_H_3_ can be formed easily from the breakage of C–C in 1,3-butadiene structure, but difficultly from the corresponding reactions of butane molecules.

## Conflicts of interest

There are no conflicts to declare.

## Supplementary Material

RA-011-D0RA08744K-s001
