# Supplementary material for: Formation pathways of polycyclic aromatic hydrocarbons (PAHs) in butane or butadiene flames
Source: RSC Adv. 2021 Feb 2;11(10):5629–42. doi: 10.1039/d0ra08744k (PMC8694769; doi:10.1039/d0ra08744k)
Supplement: RA-011-D0RA08744K-s001 [file RA-011-D0RA08744K-s001.pdf]

Electronic Supplementary Material (ESI) for RSC Advances.
This journal is © The Royal Society of Chemistry 2021

Supporting Information

Investigation of Polycyclic Aromatic Hydrocarbons (PAHs) Formation Pathways in Butane or Butadiene Flames

Tingting Zhang[1], Guizhi Mu[1], Shourong Zhang[2], Jialin Hou[1,*]

1 School of Mechanical and Electrical Engineering, Shandong Agricultural University, Taian 271018, P. R. China

*Corresponding author: jialinhou@163.com (Jialin Hou)

Table of Contents **Pages**

Table S1 Barrier heights and reaction energies for steps involved in pyrene formation pathway from phenanthrene via C_2H_3 addition computed at the G3(MP2, CC) level. 3

Table S2 Bibbs free energy barriers and reaction energies for steps involved in pyrene formation pathway from phenanthrene via C_2H_3 addition computed at the G3(MP2, CC) level. 4

Table S3 Enthalpy barriers and reaction energies for steps involved in pyrene formation pathway from phenanthrene via C_2H_3 addition computed at the G3(MP2, CC) level. 5

Table S4 Comparison of G3(MP2, CC) barrier heights for some steps optimized with B3LYP or CAM-B3LYP functionals. 6

Table S5 The molecular geometries (in Å), vibrational frequencies (in cm-1), moments of inertia (in amu) and zero-point vibrational energies (ZPE, in Hartree) of the chemical species (CS) and the transition states (TS) obtained at B3LYP/6-311++G(d, p). 7

Table S1 Barrier heights and reaction energies for steps involved in pyrene formation pathway from phenanthrene via C_2H_3 addition computed at the G3(MP2, CC) level.

No.	Reaction	Barrier/ (kcal/mol)	Reaction heat/ (kcal/mol)
R1	2CS1=CS2	14.3	-119.7
R2	CS2+H=CS3+H_2	22.4	13.1
Ra	CS2+O=CS3+OH	292.3	16.7
Rb	CS2+OH=CS3+H_2O	436.5	0.5
R3	CS3+C_2H_3=CS4	3.8	-116.2
R4	CS4+H=CS5+H_2	23.6	13.0
R5	CS5=CS6	7.3	-39.7
R6	CS6=CS7+H	27.9	16.5
R7	CS8+C_4H_4=CS9	3.4	-48.1
R8	CS9=CS10	39.1	14.3
R9	CS10=CS11	38.6	2.4
R10	CS11=CS12	0.4	-65.1
R11	CS12=CS7+H	40.5	28.8
R13	CS13+C_2H_3=CS14	5.4	-113.0
R14	CS14+H=CS15+H_2	31.0	11.9
R15	CS15=CS16	5.3	-53.2
R16	CS16=CS17+H	38.2	25.0
R17	CS18+C_4H_4=CS19	0.4	-51.0
R18	CS19=CS20	39.0	11.6
R19	CS20=CS21	7.9	-50.4
R20	CS21=CS22	37.3	0.7
R21	CS22=CS23	7.1	-38.7
R22	CS23=CS17+H	24.2	8.4
R23	CS24+C_4H_4=CS25	4.2	-48.2
R24	CS25=CS26	33.3	-47.4
R25	CS26=CS27		2.2
R26	CS27=CS28	40.7	13.6
R27	CS28=CS29	1.9	-73.8
R28	CS29=CS17+H	41.8	30.9

Table S2 Bibbs free energy barriers and reaction energies for steps involved in pyrene formation pathway from phenanthrene via C_2H_3 addition computed at the G3(MP2, CC) level.

No.	Reaction	Barrier/ (kcal/mol)	Reaction heat/ (kcal/mol)
R1	2CS1=CS2	25.4	-101.9
R2	CS2+H=CS3+H_2	26.8	10.0
Ra	CS2+O=CS3+OH	294.5	11.9
Rb	CS2+OH=CS3+H_2O	442.1	-0.7
R3	CS3+C_2H_3=CS4	15.1	-97.7
R4	CS4+H=CS5+H_2	28.0	9.8
R5	CS5=CS6	8.2	-37.1
R6	CS6=CS7+H	23.9	5.6
R7	CS8+C_4H_4=CS9	14.2	-34.2
R8	CS9=CS10	37.1	15.3
R9	CS10=CS11	36.2	2.6
R10	CS11=CS12	0.7	-61.6
R11	CS12=CS7+H	35.8	17.3
R13	CS13+C_2H_3=CS14	16.5	-94.3
R14	CS14+H=CS15+H_2	35.5	8.6
R15	CS15=CS16	5.7	-50.9
R16	CS16=CS17+H	33.8	17.8
R17	CS18+C_4H_4=CS19	9.6	-37.2
R18	CS19=CS20	37.0	12.7
R19	CS20=CS21	9.1	-46.9
R20	CS21=CS22	35.0	1.8
R21	CS22=CS23	7.5	-36.2
R22	CS23=CS17+H	20.2	-2.8
R23	CS24+C_4H_4=CS25	9.6	-34.1
R24	CS25=CS26	30.8	-47.4
R25	CS26=CS27	-	6.2
R26	CS27=CS28	38.2	14.7
R27	CS28=CS29	2.2	-70.8
R28	CS29=CS17+H	37.2	14.5

Table S3 Enthalpy barriers and reaction energies for steps involved in pyrene formation pathway from phenanthrene via C_2H_3 addition computed at the G3(MP2, CC) level.

No.	Reaction	Barrier/ (kcal/mol)	Reaction heat/ (kcal/mol)
R1	2CS1=CS2	14.4	-115.8
R2	CS2+H=CS3+H_2	20.4	11.7
Ra	CS2+O=CS3+OH	287.5	14.3
Rb	CS2+OH=CS3+H_2O	443.8	0.6
R3	CS3+C_2H_3=CS4	4.4	-111.3
R4	CS4+H=CS5+H_2	21.5	11.6
R5	CS5=CS6	6.4	-39.1
R6	CS6=CS7+H	23.4	12.0
R7	CS8+C_4H_4=CS9	3.1	-46.1
R8	CS9=CS10	36.1	15.1
R9	CS10=CS11	35.6	2.4
R10	CS11=CS12	-0.2	-64.4
R11	CS12=CS7+H	35.9	24.4
R13	CS13+C_2H_3=CS14	5.9	-108.1
R14	CS14+H=CS15+H_2	28.8	10.4
R15	CS15=CS16	4.5	-52.5
R16	CS16=CS17+H	33.6	20.5
R17	CS18+C_4H_4=CS19	0.7	-49.1
R18	CS19=CS20	36.0	12.4
R19	CS20=CS21	7.0	-49.7
R20	CS21=CS22	33.7	1.1
R21	CS22=CS23	6.1	-38.0
R22	CS23=CS17+H	20.1	4.3
R23	CS24+C_4H_4=CS25	-0.5	-46.2
R24	CS25=CS26	29.7	-47.0
R25	CS26=CS27	-	3.4
R26	CS27=CS28	37.5	14.2
R27	CS28=CS29	0.8	-73.0
R28	CS29=CS17+H	37.2	26.3

Table S4 Comparison of G3(MP2, CC) barrier heights for some steps optimized with B3LYP or CAM-B3LYP functionals.

No.	Reaction	Barrier/ (kcal/mol) B3LYP	Barrier/ (kcal/mol) G3(MP2,CC) //B3LYP	Barrier/ (kcal/mol) cam-B3LYP	Barrier/ (kcal/mol) G3(MP2,CC) //CAM-B3LYP
R6	CS6=CS7+H	32.0	27.9	34.2	31.5
R7	CS8+C_4H_4=CS9	2.0	3.4	2.3	2.8
R10	CS11=CS12	2.8	0.4	1.4	2.4
R16	CS16=CS17+H	32.9	38.2	35.4	38.2
R18	CS19=CS20	38.7	39.0	40.3	33.2
R21	CS22=CS23	5.8	7.1	7.2	9.2
R22	CS23=CS17+H	18.3	24.2	20.6	19.2
R24	CS25=CS26	23.1	33.3	28.4	34.2

Table S5 The molecular geometries (in Å), vibrational frequencies (in cm^{-1}), moments of inertia (in amu) and zero-point vibrational energies (ZPE, in Hartree) of the chemical species (CS) and the transition states (TS) obtained at B3LYP/6-311++G(d, p).

Species	ZPE, Hartree	i	Ii	Cartesian coordinates, angstroms				vi, cm^{-1}
				Atom	X	Y	Z	
H_2	0.010066	A	1811.1983	H	0	0	0.3000	4418
				H	0	0	-0.3000	
C_2H_2	0.026984	A	0.0000	C	0	0	0.6006	646 646 772 772
		B	50.7186	H	0	0	1.6706	2062 3420
		C	50.7186	C	0	0	-0.6006	
				H	0	0	-1.6706	
C_2H_3	0.036309	A	7.5985	C	0.0616	0.7587	0.0000	708 817 923 1041
		B	55.3210	H	-0.8661	1.2918	0.0000	1390 1645 3037 3135
		C	62.9195	C	0.0616	-0.5965	0.0000	3238
				H	-0.8629	-1.1352	0.0000	
				H	0.9893	-1.1297	0.0000	
C_4H_4	0.060916	A	34.8795	C	-0.4546	1.8603	0.0000	226 321 558 653
		B	381.8075	H	-0.8651	2.8403	0.0000	686 703 891 956
		C	416.6870	C	0.0000	0.7438	0.0000	1006 1110 1320 1442
				C	0.5783	-0.5558	0.0000	1662 2199 3137 3147
				H	1.6650	-0.5911	0.0000	3236 3476
				C	-0.1204	-1.6971	0.0000	
				H	-1.2039	-1.7004	0.0000	
				H	0.3842	-2.6556	0.0000	
CS1	0.086921	A	286.7623	C	-1.2245	-0.7712	0.0000	3187 3176 3174 3161
		B	321.31287	C	0.0000	-1.3956	0.0000	3155 1626 1570 1467
		C	608.07517	C	1.2245	-0.7712	0.0000	1460 1324 1301 1175
				C	1.2124	0.6316	0.0000	1174 1071 1048 1014
				C	0.0000	1.3225	0.0000	986 983 961 891
				C	-1.2124	0.6316	0.0000	812 718 665 619
				H	-2.1583	-1.3223	0.0000	600 424 398
				H	2.1583	-1.3223	0.0000	
				H	2.1510	1.1761	0.0000	
				H	0.0000	2.4065	0.0000	
				H	-2.1510	1.1761	0.0000	
CS2	0.180770	A	629.25887	C	-0.7428	0.0000	0.0000	65 93 125 267
P2		B	3309.93836	C	-1.4638	1.1272	0.4216	312 367 413 419
		C	3783.32350	C	-2.8563	1.1275	0.4218	502 556 625 628
				C	-3.5591	0.0000	0.0000	639 710 713 753
				C	-2.8563	-1.1275	-0.4218	755 795 855 855
				C	-1.4638	-1.1272	-0.4216	922 939 984 984
				C	0.7428	0.0000	0.0000	1001 1001 1010 1017
				C	1.4638	1.1272	-0.4216	1025 1052 1066 1100

				C	2.8563	1.1275	-0.4218	1105 1182 1182 1202
				C	3.5591	0.0000	0.0000	1209 1297 1301 1327
				C	2.8563	-1.1275	0.4218	1354 1358 1459 1487
				C	1.4638	-1.1272	0.4216	1514 1536 1607 1622
				H	-0.9284	2.0012	0.7748	1640 1642 3159 3160
				H	-3.3929	2.0069	0.7606	3165 3167 3174 3178
				H	-4.6432	0.0000	0.0000	3180 3183 3189 3191
				H	-3.3929	-2.0069	-0.7606	
				H	-0.9284	-2.0012	-0.7748	
				H	0.9284	2.0012	-0.7748	
				H	3.3929	2.0069	-0.7606	
				H	4.6432	0.0000	0.0000	
				H	3.3929	-2.0069	0.7606	
				H	0.9284	-2.0012	0.7748	
CS3	0.167635	A	608.90116	C	-0.7395	0.0027	0.0005	49 91 130 251
		B	3291.26432	C	-1.4852	-1.1235	-0.3017	314 381 413 419
		C	3822.68754	C	-2.8516	-1.2271	-0.3402	483 554 612 625
				C	-3.5805	-0.0701	-0.0262	639 693 705 741
				C	-2.9000	1.1026	0.3016	751 781 851 853
				C	-1.5071	1.1428	0.3180	929 943 982 984
				C	0.7432	0.0015	-0.0033	987 1000 1013 1024
				C	1.4472	-1.1788	0.2792	1048 1065 1100 1121
				C	2.8383	-1.1974	0.2805	1175 1182 1204 1239
				C	3.5566	-0.0359	-0.0017	1300 1319 1323 1356
				C	2.8695	1.1419	-0.2904	1432 1472 1481 1527
				C	1.4771	1.1603	-0.2934	1567 1614 1630 1641
				H	-3.3540	-2.1523	-0.6006	3158 3161 3165 3168
				H	-4.6649	-0.0936	-0.0358	3174 3177 3183 3187
				H	-3.4601	1.9955	0.5554	3191
				H	-1.0025	2.0611	0.5987	
				H	0.8923	-2.0815	0.5090	
				H	3.3629	-2.1192	0.5067	
				H	4.6406	-0.0496	-0.0005	
				H	3.4182	2.0478	-0.5233	
				H	0.9588	2.0778	-0.5469	
CS4	0.213398	A	1472.69757	C	-0.9561	-0.2947	0.0345	43 81 88 109
		B	3566.13354	C	-1.5612	0.6486	0.8772	186 234 289 315
		C	4668.95155	C	-2.9444	0.8126	0.8874	325 416 427 474
				C	-3.7499	0.0389	0.0529	542 567 583 631
				C	-3.1617	-0.9025	-0.7898	635 666 715 733
				C	-1.7786	-1.0696	-0.7959	760 784 798 811
				C	0.5188	-0.5104	0.0432	859 890 935 937
				C	2.8161	0.2439	-0.1257	969 988 996 1004
				C	3.2777	-1.0396	0.1347	1013 1023 1031 1042

				C	2.3650	-2.0728	0.3425	1057 1079 1099 1126
				C	1.0021	-1.8033	0.2911	1181 1186 1203 1219
				H	-0.9432	1.2461	1.5374	1282 1292 1318 1323
				H	-3.3936	1.5418	1.5526	1340 1354 1449 1463
				H	-4.8263	0.1687	0.0597	1480 1506 1528 1597
				H	-3.7792	-1.5056	-1.4464	1614 1635 1640 1678
				H	-1.3264	-1.7966	-1.4616	3135 3154 3159 3162
				H	3.5288	1.0334	-0.3336	3166 3171 3176 3181
				H	4.3436	-1.2369	0.1587	3182 3190 3190 3218
				H	2.7116	-3.0804	0.5423	
				H	0.2896	-2.6010	0.4695	
				C	1.4435	0.5400	-0.1706	
				C	0.9887	1.9059	-0.4957	
				H	0.0160	1.9768	-0.9731	
				C	1.6583	3.0372	-0.2539	
				H	2.6144	3.0510	0.2587	
				H	1.2556	3.9968	-0.5556	
CS5	0.200103	A	1338.50498	C	-0.8151	-0.3351	0.0303	42 81 90 121
		B	3546.31403	C	-1.5988	-0.9803	-0.9044	192 212 272 298
		C	4390.93571	C	-2.9717	-1.0319	-0.9363	316 345 415 439
				C	-3.6485	-0.3750	0.1001	470 495 535 559
				C	-2.9201	0.2858	1.0915	607 622 631 682
				C	-1.5282	0.3090	1.0614	708 737 742 760
				C	0.6762	-0.3886	0.0010	765 832 858 870
				C	2.8956	0.5698	0.1038	947 958 965 984
				C	3.4694	-0.6951	0.1346	985 988 1011 1045
				C	2.6503	-1.8198	0.0789	1060 1090 1117 1131
				C	1.2700	-1.6575	0.0195	1174 1175 1220 1237
				H	-3.5130	-1.5540	-1.7178	1255 1309 1316 1318
				H	-4.7328	-0.3869	0.1307	1392 1430 1434 1455
				H	-3.4433	0.7851	1.8993	1468 1493 1559 1565
				H	-0.9754	0.8265	1.8380	1600 1625 3092 3117
				H	3.5353	1.4460	0.1273	3158 3161 3165 3170
				H	4.5465	-0.8007	0.1971	3175 3176 3178 3186
				H	3.0786	-2.8155	0.0987	3190
				H	0.6249	-2.5288	0.0114	
				C	1.5057	0.7582	0.0088	
				C	1.0429	2.1562	-0.1213	
				H	1.7059	2.8850	0.3411	
				C	-0.0133	2.6188	-0.7951	
				H	-0.7077	1.9717	-1.3156	
				H	-0.2053	3.6845	-0.8475	
CS6	0.202383	A	1161.92643	C	-3.5803	-0.3363	-0.0001	31 90 170 221
		B	3315.31804	C	-2.8736	0.8461	-0.0001	249 276 396 426

		C	4466.38581	C	-1.4465	0.8610	-0.0001	432 448 495 498
				C	-0.7436	-0.3951	0.0000	548 563 630 672
				C	-1.4972	-1.5728	0.0001	696 713 717 729
				C	-2.8902	-1.5589	0.0001	761 782 818 850
				C	-0.7510	2.0730	-0.0001	863 864 927 940
				C	0.7341	-0.3920	0.0000	962 974 987 1007
				C	1.4445	0.8270	0.0001	1041 1060 1074 1113
				C	0.7330	2.1612	0.0002	1153 1175 1188 1197
				C	2.8430	0.8164	0.0000	1203 1217 1264 1297
				H	3.3740	1.7639	0.0000	1302 1330 1338 1375
				C	3.5600	-0.3725	-0.0001	1431 1447 1458 1483
				C	2.8666	-1.5839	-0.0001	1499 1533 1564 1603
				C	1.4802	-1.5877	-0.0001	1611 1642 2945 2947
				H	-1.3140	3.0008	-0.0002	3150 3157 3161 3168
				H	-4.6645	-0.3235	-0.0001	3172 3182 3185 3194
				H	-3.3969	1.7966	-0.0002	3207
				H	-0.9993	-2.5338	0.0002	
				H	-3.4373	-2.4944	0.0002	
				H	1.0703	2.7503	-0.8681	
				H	4.6439	-0.3580	-0.0001	
				H	3.4074	-2.5236	-0.0001	
				H	0.9704	-2.5421	-0.0001	
				H	1.0700	2.7499	0.8689	
CS7	0.193561	A	1115.17950	C	3.5569	-0.2969	0.0000	95 100 227 242
		B	3272.40099	C	2.8347	0.8773	-0.0001	248 400 411 436
		C	4387.58048	C	1.4214	0.8653	0.0000	446 505 507 542
				C	0.7283	-0.3792	0.0000	556 599 632 722
				C	1.4979	-1.5646	0.0001	725 726 746 766
				C	2.8783	-1.5281	0.0001	798 826 842 871
				C	0.6786	2.0921	0.0000	879 887 955 966
				C	-0.7283	-0.3792	0.0000	980 991 991 1016
				C	-1.4214	0.8653	0.0000	1059 1060 1114 1164
				C	-0.6786	2.0921	0.0000	1173 1185 1188 1224
				C	-2.8347	0.8773	0.0000	1241 1266 1303 1324
				H	-3.3464	1.8342	0.0001	1368 1374 1443 1449
				C	-3.5569	-0.2969	0.0000	1469 1489 1533 1560
				C	-2.8783	-1.5281	-0.0001	1606 1640 1652 1657
				C	-1.4979	-1.5646	-0.0001	3155 3159 3160 3169
				H	1.2288	3.0273	0.0000	3170 3175 3183 3186
				H	4.6407	-0.2733	-0.0001	3193 3206
				H	3.3464	1.8342	-0.0001	
				H	1.0055	-2.5284	0.0002	
				H	3.4406	-2.4551	0.0001	
				H	-1.2288	3.0273	0.0001	

				H	-4.6407	-0.2733	0.0000	
				H	-3.4406	-2.4551	-0.0001	
				H	-1.0055	-2.5284	-0.0001	
CS8	0.133723	A	573.07843	C	-2.3959	0.7952	0.0000	174 190 368 386
		B	1414.98779	C	-1.2358	1.4938	0.0000	471 486 510 520
		C	1988.06621	C	-0.0174	0.7449	0.0000	607 627 744 753
				C	-0.0869	-0.6867	0.0000	766 792 805 845
				C	-1.3560	-1.3274	0.0000	884 935 956 974
				C	-2.5256	-0.5934	0.0000	995 1038 1048 1140
				H	1.3050	2.4560	0.0000	1160 1172 1207 1249
				H	-1.2023	2.5785	0.0000	1276 1333 1384 1393
				C	1.2553	1.3722	0.0000	1455 1466 1527 1588
				C	1.1250	-1.4271	0.0000	1617 1652 3155 3159
				H	-1.3961	-2.4125	0.0000	3160 3164 3176 3180
				C	2.3429	-0.7903	0.0000	3188
				C	2.4078	0.6227	0.0000	
				H	1.0741	-2.5110	0.0000	
				H	3.2594	-1.3693	0.0000	
				H	3.3742	1.1146	0.0000	
				H	-3.4944	-1.0797	0.0000	
CS9	0.198321	A	1126.38614	C	-0.8745	0.9515	-0.0716	31 49 102 150
		B	5029.65219	C	-0.2940	-0.3023	-0.1278	182 217 224 318
		C	6053.80906	C	1.1086	-0.4726	-0.0621	349 410 419 482
				C	1.9472	0.6811	0.0658	504 524 529 544
				C	1.3335	1.9588	0.1221	581 641 649 729
				C	-0.0310	2.0897	0.0559	750 754 776 779
				H	1.0833	-2.6314	-0.2151	809 842 874 908
				H	-0.9236	-1.1812	-0.2237	917 935 936 966
				C	1.7176	-1.7564	-0.1185	978 979 993 1039
				C	3.3538	0.5058	0.1317	1083 1147 1172 1176
				H	1.9611	2.8387	0.2190	1184 1190 1226 1263
				C	3.9104	-0.7496	0.0741	1288 1315 1383 1393
				C	3.0829	-1.8917	-0.0523	1402 1416 1469 1489
				H	3.9861	1.3825	0.2287	1498 1542 1604 1637
				H	4.9866	-0.8707	0.1252	1663 1884 3059 3096
				H	3.5338	-2.8768	-0.0968	3149 3156 3157 3161
				C	-2.3375	1.1582	-0.1389	3164 3174 3175 3187
				H	-2.6609	2.2005	-0.0779	3250
				C	-3.2589	0.2378	-0.2598	
				C	-4.2242	-0.6941	-0.3953	
				H	-4.5089	-0.9564	-1.4150	
				C	-4.8815	-1.3447	0.6487	
				H	-4.6444	-1.1281	1.6825	
				H	-5.6456	-2.0821	0.4414	

				H	-0.4824	3.0754	0.1007	
CS10	0.199581	A	836.22404	C	-0.9095	0.4789	-0.0686	49 55 86 146
		B	5700.43574	C	-0.0424	-0.5785	-0.0281	178 193 211 303
		C	6505.05449	C	1.3494	-0.5437	0.0049	308 411 435 473
				C	1.9451	0.7691	-0.0072	497 516 522 580
				C	1.0878	1.9013	-0.0495	626 640 665 750
				C	-0.2775	1.7676	-0.0789	759 768 777 816
				H	1.7351	-2.6716	0.0600	867 891 924 934
				H	-2.5236	-1.7063	-0.2578	956 969 970 990
				C	2.1913	-1.6888	0.0501	991 1003 1027 1037
				C	3.3589	0.8693	0.0246	1081 1142 1167 1172
				H	1.5331	2.8904	-0.0579	1201 1235 1263 1313
				C	4.1459	-0.2575	0.0674	1330 1349 1360 1365
				C	3.5568	-1.5451	0.0808	1390 1444 1452 1490
				H	3.8122	1.8552	0.0145	1516 1574 1623 1644
				H	5.2257	-0.1638	0.0912	1663 1687 3123 3133
				H	4.1924	-2.4227	0.1153	3140 3144 3152 3158
				C	-2.3670	0.3750	-0.0985	3169 3175 3182 3192
				H	-2.9022	1.3211	-0.0798	3220
				C	-3.0711	-0.7705	-0.1687	
				C	-4.5284	-0.8759	-0.1714	
				H	-4.9303	-1.7629	-0.6570	
				C	-5.3913	-0.0141	0.3826	
				H	-5.0588	0.8588	0.9343	
				H	-6.4612	-0.1697	0.3119	
				H	-0.9097	2.6499	-0.1102	
CS11	0.199755	A	1256.98257	C	1.1471	0.9793	0.0733	35 69 92 127
		B	3925.35874	C	0.4684	-0.2078	0.0620	180 188 244 322
		C	5150.97391	C	-0.9151	-0.3962	0.0225	384 418 427 467
				C	-1.7183	0.7974	-0.0182	500 519 524 594
				C	-1.0580	2.0569	-0.0093	623 666 697 748
				C	0.3086	2.1480	0.0336	761 772 783 800
				H	-0.9449	-2.5583	0.0415	831 871 895 931
				H	4.6030	0.7346	-0.1269	938 958 971 990
				C	-1.5568	-1.6643	0.0181	992 999 1021 1038
				C	-3.1284	0.6642	-0.0622	1089 1139 1161 1173
				H	-1.6590	2.9597	-0.0355	1194 1236 1262 1276
				C	-3.7199	-0.5779	-0.0626	1334 1352 1362 1398
				C	-2.9277	-1.7499	-0.0215	1439 1445 1471 1492
				H	-3.7374	1.5618	-0.0968	1515 1576 1617 1642
				H	-4.8001	-0.6638	-0.0968	1663 1676 3111 3123
				H	-3.4096	-2.7210	-0.0251	3139 3144 3151 3157
				C	2.5971	1.1675	0.1014	3169 3173 3181 3191
				H	2.8923	2.2068	0.2217	3224

				C	3.6103	0.2913	-0.0806	
				C	3.5960	-1.1503	-0.3045	
				H	4.3756	-1.5077	-0.9760	
				C	2.7901	-2.0621	0.2558	
				H	2.0479	-1.7988	0.9987	
				H	2.8854	-3.1132	0.0069	
				H	0.7865	3.1231	0.0413	
CS12	0.202323	A	1151.03759	C	1.3721	0.9092	0.0000	84 116 164 221
		B	3303.34178	C	0.6949	-0.3216	0.0000	231 294 404 417
		C	4443.54346	C	-0.7319	-0.3484	0.0000	445 472 493 530
				C	-1.4641	0.8840	0.0000	539 542 604 653
				C	-0.7455	2.1143	0.0000	670 702 715 747
				C	0.6174	2.1254	0.0000	765 789 832 836
				H	-0.9542	-2.5097	0.0000	860 866 937 953
				H	4.6477	-0.1569	-0.0001	959 967 978 988
				C	-1.4760	-1.5612	0.0000	1003 1051 1071 1121
				C	-2.8777	0.8551	0.0000	1167 1174 1181 1200
				H	-1.3037	3.0448	0.0000	1204 1228 1235 1287
				C	-3.5654	-0.3386	0.0000	1314 1344 1382 1406
				C	-2.8532	-1.5560	0.0000	1415 1440 1444 1458
				H	-3.4166	1.7971	0.0000	1487 1542 1564 1588
				H	-4.6494	-0.3456	0.0000	1633 1645 2936 2940
				H	-3.3952	-2.4952	0.0000	3154 3155 3158 3168
				C	2.8009	0.9543	0.0000	3171 3174 3184 3184
				H	3.2925	1.9202	0.0000	3196
				C	3.5649	-0.2274	0.0000	
				C	2.9669	-1.4559	0.0001	
				H	3.5671	-2.3594	0.0002	
				C	1.4786	-1.6170	0.0000	
				H	1.1816	-2.2295	0.8676	
				H	1.1816	-2.2295	-0.8676	
				H	1.1572	3.0666	0.0000	
CS13	0.180474	A	1093.99344	C	3.5598	-0.3684	0.0000	99 115 221 223
		B	3259.66075	C	2.8557	0.8188	0.0000	247 408 409 446
		C	4353.65419	C	1.4412	0.8411	0.0000	450 494 499 554
				C	0.7196	-0.3940	0.0000	556 586 628 705
				C	1.5076	-1.5413	0.0000	720 724 741 769
				C	2.8667	-1.6041	0.0000	794 826 835 880
				C	0.7007	2.0700	0.0000	887 888 965 968
				C	-0.7284	-0.3893	0.0000	983 995 1011 1049
				C	-1.4141	0.8555	0.0000	1064 1098 1162 1171
				C	-0.6586	2.0759	0.0000	1179 1189 1229 1242
				C	-2.8273	0.8611	0.0000	1281 1313 1354 1367
				H	-3.3469	1.8138	0.0000	1423 1430 1448 1474

				C	-3.5405	-0.3198	0.0000	1512 1531 1583 1633
				C	-2.8599	-1.5517	0.0000	1647 1652 3155 3159
				C	-1.4798	-1.5835	0.0000	3159 3166 3169 3174
				H	1.2535	3.0038	0.0000	3177 3182 3188
				H	4.6445	-0.3634	0.0000	
				H	3.3892	1.7634	0.0000	
				H	3.4063	-2.5447	0.0000	
				H	-1.2004	3.0160	0.0000	
				H	-4.6245	-0.3011	0.0000	
				H	-3.4223	-2.4787	0.0000	
				H	-0.9541	-2.5319	0.0000	
CS14	0.226429	A	2122.34389	C	-3.6359	0.8337	0.0397	58 97 130 155
		B	3554.41093	C	-3.2415	-0.4781	-0.1145	226 240 276 312
		C	5616.70353	C	-1.8763	-0.8396	-0.0992	338 403 416 476
				C	-0.8705	0.1684	0.0230	500 509 514 541
				C	-1.3175	1.4940	0.2443	569 587 626 682
				C	-2.6606	1.8210	0.2474	703 727 742 758
				C	-1.5033	-2.2215	-0.1417	784 799 812 846
				C	0.5386	-0.2210	-0.0183	855 883 923 939
				C	0.8444	-1.6158	0.0489	969 979 985 988
				C	-0.2054	-2.5907	-0.0225	996 1011 1045 1055
				C	2.1813	-2.0538	0.1546	1071 1120 1153 1174
				H	2.3763	-3.1189	0.2203	1185 1196 1225 1241
				C	3.2192	-1.1494	0.1554	1254 1289 1319 1325
				C	2.9404	0.2111	-0.0140	1345 1362 1414 1441
				C	1.6374	0.6948	-0.1197	1451 1468 1480 1528
				H	-2.2865	-2.9672	-0.2278	1556 1602 1624 1645
				H	-4.6878	1.0959	0.0317	1659 1674 3130 3138
				H	-3.9823	-1.2627	-0.2289	3156 3159 3163 3170
				H	-0.6011	2.2724	0.4555	3176 3178 3185 3191
				H	-2.9588	2.8479	0.4269	3217 3230
				H	0.0706	-3.6397	-0.0009	
				H	4.2466	-1.4861	0.2339	
				H	3.7596	0.9112	-0.1242	
				C	1.4827	2.1265	-0.4623	
				H	0.7597	2.3503	-1.2425	
				C	2.1899	3.1322	0.0601	
				H	2.8983	2.9786	0.8673	
				H	2.0675	4.1477	-0.2985	
CS15	0.212851	A	2106.73692	C	3.2744	-0.9885	0.0086	60 82 109 156
		B	3457.49098	C	2.2894	-1.9515	0.0522	225 231 246 301
		C	5520.62916	C	0.9269	-1.5861	0.0137	345 405 421 467
				C	0.5639	-0.2113	-0.0662	491 503 516 542
				C	1.5941	0.7733	-0.1430	565 575 624 675

				C	2.9235	0.3646	-0.0992	688 720 724 754
				C	-0.0952	-2.5929	0.0581	773 799 809 841
				C	-0.8462	0.1289	-0.0385	849 888 923 940
				C	-1.8393	-0.9041	0.0037	967 977 981 987
				C	-1.4142	-2.2717	0.0415	1005 1011 1054 1065
				C	-3.2090	-0.5546	0.0277	1107 1143 1172 1187
				H	-3.9449	-1.3511	0.0556	1192 1228 1250 1273
				C	-3.6210	0.7634	0.0250	1300 1321 1344 1355
				C	-2.6602	1.8013	0.0028	1407 1426 1443 1448
				C	-1.3510	1.4261	-0.0246	1471 1505 1523 1585
				H	0.2143	-3.6317	0.1054	1623 1641 1650 1666
				H	4.3205	-1.2723	0.0360	3128 3134 3156 3157
				H	2.5486	-3.0031	0.1147	3161 3167 3174 3176
				H	3.7014	1.1142	-0.1846	3180 3189 3218
				H	-2.1715	-3.0482	0.0716	
				H	-4.6776	1.0085	0.0472	
				H	-2.9650	2.8421	0.0128	
				C	1.2930	2.2096	-0.3171	
				H	0.5734	2.4609	-1.0922	
				C	1.8693	3.1998	0.3715	
				H	2.5717	3.0041	1.1751	
				H	1.6381	4.2376	0.1614	
CS16	0.214991	A	1832.19022	C	3.5444	-0.0301	-0.0004	63 120 184 211
		B	3293.16172	C	2.8647	1.1818	-0.0003	238 288 352 402
		C	5114.48660	C	1.4465	1.2287	-0.0001	436 448 489 499
				C	0.7215	-0.0209	-0.0001	507 528 549 561
				C	1.4311	-1.2441	-0.0002	582 625 685 685
				C	2.8473	-1.2331	-0.0003	713 732 758 769
				C	0.7510	2.4377	-0.0001	793 802 816 836
				C	-0.7170	-0.0219	0.0001	871 899 937 947
				C	-1.4533	1.1910	0.0001	971 978 985 1002
				C	-0.7374	2.5258	0.0000	1048 1093 1109 1113
				C	-2.8381	1.1460	0.0003	1168 1188 1193 1197
				H	-3.4008	2.0748	0.0004	1210 1226 1256 1257
				C	-3.5273	-0.0770	0.0004	1319 1339 1362 1379
				C	-2.8264	-1.2661	0.0003	1415 1437 1439 1455
				C	-1.4165	-1.2649	0.0002	1459 1477 1522 1537
				C	-0.6646	-2.4848	0.0001	1584 1602 1636 1649
				C	0.6950	-2.4754	-0.0001	2952 2953 3152 3155
				H	1.2498	-3.4079	-0.0001	3157 3161 3163 3170
				H	-1.2069	-3.4247	0.0001	3174 3183 3184
				H	1.3112	3.3672	-0.0001	
				H	4.6289	-0.0366	-0.0005	
				H	3.4166	2.1157	-0.0003	

				H	3.3819	-2.1767	-0.0004	
				H	-1.0711	3.1154	0.8688	
				H	-4.6116	-0.0828	0.0005	
				H	-3.3527	-2.2148	0.0004	
				H	-1.0712	3.1154	-0.8687	
CS17	0.206163	A	1782.55145	C	3.5194	0.0000	0.0003	97 150 210 246
		B	3244.15401	C	2.8296	-1.2091	0.0003	260 358 402 411
		C	5026.70546	C	1.4273	-1.2343	0.0002	462 499 505 507
				C	0.7128	0.0000	0.0001	507 538 553 581
				C	1.4273	1.2343	0.0001	594 687 703 718
				C	2.8296	1.2091	0.0003	748 752 766 809
				C	0.6795	-2.4612	0.0001	811 814 833 854
				C	-0.7128	0.0000	-0.0001	902 916 974 979
				C	-1.4273	-1.2343	-0.0001	980 982 989 1014
				C	-0.6795	-2.4612	0.0000	1089 1112 1125 1164
				C	-2.8296	-1.2091	-0.0003	1166 1197 1203 1230
				H	-3.3758	-2.1460	-0.0003	1261 1262 1266 1342
				C	-3.5194	0.0000	-0.0004	1350 1399 1425 1432
				C	-2.8296	1.2091	-0.0003	1456 1458 1481 1512
				C	-1.4273	1.2343	-0.0002	1532 1592 1624 1632
				C	-0.6795	2.4612	-0.0001	1642 1667 3161 3161
				C	0.6795	2.4612	0.0001	3164 3164 3170 3172
				H	1.2271	3.3974	0.0002	3180 3180 3188 3189
				H	-1.2271	3.3974	-0.0001	
				H	1.2271	-3.3974	0.0002	
				H	4.6031	0.0000	0.0004	
				H	3.3758	-2.1460	0.0003	
				H	3.3758	2.1460	0.0004	
				H	-1.2271	-3.3974	0.0000	
				H	-4.6031	0.0000	-0.0005	
				H	-3.3758	2.1460	-0.0004	
CS18	0.142704	A	1175.87563	C	-1.7281	-1.9791	0.0001	103 138 176 207
		B	1892.59436	C	-0.4048	-1.6894	-0.0001	356 363 436 445
		C	3068.46999	C	-0.0351	-0.3105	0.0000	479 490 551 568
				C	-1.0702	0.6798	0.0002	587 632 644 696
				C	-2.4325	0.2728	0.0003	701 744 768 791
				C	-2.7785	-1.0634	0.0002	827 862 868 928
				H	0.3656	-2.4521	-0.0002	960 987 1029 1053
				C	1.3350	0.1175	-0.0002	1094 1150 1191 1215
				C	-0.7142	2.0534	0.0002	1241 1277 1325 1355
				H	-3.2054	1.0352	0.0004	1386 1446 1471 1512
				H	-3.8163	-1.3768	0.0003	1592 1612 1636 2195
				C	0.6050	2.4394	0.0001	3157 3165 3177 3180
				C	1.6291	1.4730	-0.0001	3180 3194 3475

				H	-1.5037	2.7974	0.0004	
				H	0.8668	3.4912	0.0001	
				H	2.6664	1.7852	-0.0002	
				C	2.3933	-0.8388	-0.0003	
				C	3.2911	-1.6432	-0.0005	
				H	4.0860	-2.3483	-0.0006	
CS19	0.207231	A	2107.56530	C	-0.9778	-1.3211	-0.0987	24 45 86 115
		B	5081.48293	C	-0.3546	-0.0853	-0.1017	127 158 187 222
		C	7076.44421	C	1.0507	0.0338	-0.0244	230 312 340 386
				C	1.8465	-1.1533	0.0592	403 448 454 508
				C	1.1887	-2.4103	0.0606	517 528 551 580
				C	-0.1781	-2.4928	-0.0158	586 593 636 672
				H	-0.9517	0.8168	-0.1637	695 712 734 751
				C	1.7175	1.3087	-0.0262	758 785 819 854
				C	3.2578	-1.0473	0.1381	872 912 925 927
				H	1.7877	-3.3128	0.1237	935 953 973 985
				H	-0.6634	-3.4632	-0.0133	1047 1082 1094 1175
				C	3.8704	0.1834	0.1348	1179 1190 1199 1231
				C	3.1005	1.3608	0.0525	1253 1289 1308 1351
				H	3.8479	-1.9555	0.2015	1389 1402 1419 1472
				H	4.9500	0.2577	0.1951	1476 1489 1537 1603
				H	3.5907	2.3267	0.0501	1626 1653 1883 2194
				C	0.9691	2.5200	-0.1085	3059 3098 3149 3158
				C	0.3427	3.5477	-0.1778	3163 3175 3180 3184
				H	-0.2071	4.4550	-0.2380	3194 3251 3475
				C	-2.4465	-1.4718	-0.1788	
				H	-2.8069	-2.5036	-0.1700	
				C	-3.3335	-0.5134	-0.2549	
				C	-4.2653	0.4569	-0.3450	
				H	-4.5349	0.7808	-1.3509	
				C	-4.9038	1.0757	0.7293	
				H	-4.6778	0.7994	1.7512	
				H	-5.6398	1.8500	0.5580	
CS20	0.208491	A	1952.88612	C	1.0036	-1.1075	0.0000	33 61 83 112
		B	5729.80452	C	0.2011	0.0019	0.0000	122 156 183 215
		C	7682.69064	C	-1.1883	0.0486	0.0000	244 290 319 372
				C	-1.8628	-1.2232	0.0000	381 452 469 491
				C	-1.0743	-2.4055	0.0000	520 535 554 580
				C	0.2967	-2.3557	0.0000	589 632 644 684
				C	-1.9619	1.2574	0.0000	694 712 747 756
				C	-3.2788	-1.2446	0.0000	781 831 870 895
				H	-1.5796	-3.3652	0.0000	924 928 959 960
				H	0.8742	-3.2752	0.0000	965 975 983 1038
				C	-4.0002	-0.0725	0.0000	1041 1088 1158 1171

				C	-3.3450	1.1757	0.0000	1191 1221 1244 1259
				H	-3.7879	-2.2027	0.0000	1293 1314 1324 1345
				H	-5.0836	-0.1012	0.0000	1358 1394 1443 1455
				H	-3.9257	2.0900	0.0000	1468 1510 1577 1621
				C	-1.3069	2.5230	0.0000	1628 1651 1687 2197
				C	-0.7596	3.5969	0.0000	3125 3132 3136 3141
				H	-0.2759	4.5428	-0.0001	3153 3163 3176 3181
				C	2.4630	-1.0866	-0.0001	3195 3223 3476
				H	2.9472	-2.0607	-0.0001	
				C	3.2339	0.0197	0.0000	
				C	4.6813	-0.0057	0.0000	
				H	5.1505	-0.9878	-0.0002	
				C	5.4575	1.0883	0.0001	
				H	5.0297	2.0857	0.0002	
				H	6.5383	1.0173	0.0000	
				H	2.7558	0.9970	0.0002	
CS21	0.211293	A	2133.63287	C	3.6769	0.3905	0.0001	37 102 134 164
		B	3475.10892	C	3.0820	-0.8837	0.0000	185 220 238 295
		C	5598.06311	C	1.6636	-1.0452	0.0000	364 381 412 470
				C	0.7977	0.0695	0.0000	496 499 502 531
				C	1.4090	1.4575	0.0001	544 586 593 621
				C	2.9038	1.5135	0.0001	649 666 681 685
				C	1.1263	-2.3685	0.0000	718 755 770 789
				C	-0.6228	-0.1286	0.0000	807 840 844 930
				C	-1.1257	-1.4797	0.0000	932 934 968 976
				C	-0.2158	-2.5756	-0.0001	983 993 1003 1090
				C	-2.5113	-1.7439	-0.0001	1118 1151 1179 1184
				H	-2.8384	-2.7783	-0.0001	1199 1218 1219 1246
				C	-3.4308	-0.7205	-0.0001	1265 1307 1337 1345
				C	-2.9762	0.6025	0.0000	1387 1415 1425 1440
				C	-1.6183	0.9229	0.0000	1455 1475 1532 1570
				H	1.8148	-3.2069	0.0000	1583 1620 1644 2179
				H	4.7587	0.4739	0.0001	2984 2987 3155 3159
				H	3.7011	-1.7733	0.0000	3164 3171 3178 3181
				H	1.0330	2.0266	-0.8612	3184 3196 3473
				H	3.3595	2.4979	0.0001	
				H	-0.6211	-3.5818	-0.0001	
				H	-4.4945	-0.9271	-0.0001	
				H	-3.6906	1.4164	0.0000	
				H	1.0330	2.0266	0.8613	
				C	-1.3533	2.3286	0.0000	
				C	-1.2835	3.5332	0.0000	
				H	-1.1850	4.5910	0.0001	
CS22	0.212430	A	2081.82819	C	3.6380	0.7403	-0.0463	58 98 127 156

		B	3532.86060	C	3.1992	-0.5530	0.1419	224 239 279 319
		C	5553.36521	C	1.8233	-0.8701	0.1227	343 403 416 479
				C	0.8535	0.1674	-0.0343	507 510 516 539
				C	1.3450	1.4700	-0.2938	568 583 615 668
				C	2.6983	1.7525	-0.2946	678 725 733 756
				C	1.4027	-2.2377	0.1899	782 789 802 837
				C	-0.5677	-0.1741	0.0040	847 860 881 889
				C	-0.9209	-1.5584	-0.0480	927 971 980 988
				C	0.0951	-2.5661	0.0564	997 1002 1012 1068
				C	-2.2704	-1.9496	-0.1685	1113 1150 1174 1184
				H	-2.5027	-3.0078	-0.2236	1193 1221 1240 1241
				C	-3.2760	-1.0082	-0.1996	1264 1292 1322 1344
				C	-2.9535	0.3425	-0.0376	1362 1416 1446 1464
				C	-1.6354	0.7756	0.0896	1479 1527 1556 1601
				H	2.1591	-3.0072	0.3024	1622 1640 1648 1658
				H	4.6979	0.9678	-0.0360	3029 3157 3159 3163
				H	3.9130	-1.3578	0.2846	3170 3176 3178 3186
				H	0.6562	2.2631	-0.5407	3190 3227 3245
				H	3.0324	2.7622	-0.5052	
				H	-0.2154	-3.6055	0.0494	
				H	-4.3127	-1.3096	-0.2964	
				H	-3.7438	1.0794	0.0425	
				C	-1.4311	2.2030	0.4402	
				C	-2.1418	3.2201	0.0078	
				H	-2.1890	4.2865	0.1682	
				H	-0.6452	2.4048	1.1755	
CS23	0.214547	A	1822.34400	C	-3.5354	-0.0585	-0.0604	81 133 193 216
		B	3283.97187	C	-2.8884	1.1599	-0.0209	244 337 382 396
		C	5062.75504	C	-1.4770	1.2260	0.0261	442 457 487 498
				C	-0.7271	0.0093	0.0699	506 521 538 550
				C	-1.4143	-1.2467	-0.0157	580 650 673 679
				C	-2.7985	-1.2584	-0.0765	705 732 761 774
				C	-0.7663	2.4617	0.0189	798 811 818 844
				C	0.6829	0.0532	0.1396	849 911 946 959
				C	1.3653	1.2792	0.0476	971 978 987 990
				C	0.5998	2.4867	0.0061	1061 1073 1093 1098
				C	2.7860	1.2850	-0.0755	1141 1168 1173 1188
				H	3.3029	2.2342	-0.1595	1198 1216 1231 1255
				C	3.4996	0.0778	-0.2305	1260 1294 1334 1371
				C	2.8875	-1.1367	-0.0862	1397 1413 1418 1425
				C	1.4653	-1.2173	0.3959	1457 1476 1520 1553
				C	0.7117	-2.4634	0.0090	1584 1621 1628 1681
				C	-0.6214	-2.4653	-0.1264	2750 3148 3155 3156
				H	-1.1473	-3.3887	-0.3492	3158 3165 3169 3172

				H	1.2811	-3.3824	-0.0896	3174 3182 3184
				H	-1.3311	3.3880	-0.0015	
				H	-4.6182	-0.0962	-0.0999	
				H	-3.4577	2.0833	-0.0364	
				H	-3.3199	-2.2070	-0.1527	
				H	1.1279	3.4335	-0.0413	
				H	4.5527	0.1203	-0.4894	
				H	3.4390	-2.0617	-0.2153	
				H	1.5525	-1.2786	1.5089	
CS24	0.142679	A	1201.89511	C	-1.8608	-1.9119	0.0000	105 126 169 201
		B	1898.15438	C	-0.5478	-1.5764	-0.0001	339 364 443 453
		C	3100.04949	C	-0.0186	-0.2824	0.0000	476 489 542 577
				C	-0.9813	0.7855	0.0002	592 626 647 694
				C	-2.3661	0.4633	0.0003	702 749 757 798
				C	-2.7999	-0.8411	0.0002	812 867 883 926
				C	1.3807	0.0241	-0.0001	970 988 1020 1051
				C	-0.5109	2.1235	0.0002	1087 1164 1188 1196
				H	-3.0856	1.2749	0.0004	1238 1262 1343 1358
				H	-3.8612	-1.0657	0.0003	1398 1452 1464 1506
				C	0.8373	2.3952	0.0001	1583 1621 1651 2199
				C	1.7832	1.3503	-0.0001	3159 3163 3170 3180
				H	-1.2344	2.9321	0.0004	3182 3195 3477
				H	1.1844	3.4222	0.0001	
				H	2.8418	1.5796	-0.0002	
				C	2.3409	-1.0298	-0.0003	
				C	3.1568	-1.9166	-0.0005	
				H	3.8719	-2.7024	-0.0006	
				H	-2.1981	-2.9425	0.0000	
CS25	0.207298	A	2085.42594	C	0.8473	-1.8113	-0.0899	27 44 62 130
		B	4609.11605	C	0.5026	-0.4706	-0.1623	153 172 185 196
		C	6620.10735	C	-0.8830	-0.0939	-0.0679	235 325 344 369
				C	-1.8576	-1.1489	0.0311	392 475 479 502
				C	-1.4424	-2.5034	0.0931	515 527 547 564
				C	-0.1113	-2.8286	0.0526	586 614 635 650
				C	-1.3825	1.2604	-0.0419	686 701 744 767
				C	-3.2429	-0.8477	0.0764	775 798 811 812
				H	-2.2010	-3.2744	0.1717	844 905 922 933
				H	0.2067	-3.8638	0.1014	938 985 991 1000
				C	-3.6863	0.4494	0.0473	1046 1092 1115 1139
				C	-2.7528	1.4965	0.0010	1185 1189 1193 1230
				H	-3.9470	-1.6706	0.1389	1244 1279 1298 1351
				H	-4.7459	0.6747	0.0789	1369 1386 1420 1458
				H	-3.0953	2.5238	0.0167	1483 1490 1540 1604
				C	-0.5648	2.4333	-0.0036	1627 1641 1889 2188

				C	0.0098	3.4918	0.0568	3098 3122 3148 3163
				H	0.5512	4.4045	0.1098	3166 3177 3182 3189
				H	1.8943	-2.0768	-0.1772	3196 3249 3474
				C	1.5920	0.5120	-0.3933	
				H	1.3467	1.3852	-0.9924	
				C	2.8185	0.3913	0.0409	
				C	4.0870	0.3268	0.4950	
				H	4.2842	0.8055	1.4548	
				C	5.1560	-0.2953	-0.1503	
				H	5.0273	-0.7839	-1.1079	
				H	6.1418	-0.2952	0.2954	
CS26	0.207871	A	2164.88647	C	-0.1860	-2.3285	0.0806	31 40 52 100
		B	4570.65370	C	-0.3087	-0.9406	0.0715	122 161 193 198
		C	6706.54911	C	0.8771	-0.1147	0.0574	249 287 326 348
				C	2.1441	-0.7902	-0.0339	402 436 473 488
				C	2.2057	-2.2072	-0.0303	508 538 549 567
				C	1.0638	-2.9644	0.0421	601 637 650 673
				C	0.8866	1.3221	0.1078	693 759 770 774
				C	3.3457	-0.0437	-0.1356	801 822 843 864
				H	3.1796	-2.6811	-0.0896	901 921 937 938
				H	1.1164	-4.0469	0.0491	984 991 1000 1018
				C	3.3197	1.3271	-0.1381	1052 1102 1116 1155
				C	2.0929	1.9979	-0.0002	1189 1201 1228 1253
				H	4.2846	-0.5816	-0.2089	1271 1304 1317 1349
				H	4.2384	1.8971	-0.2170	1371 1393 1446 1459
				H	2.0785	3.0796	0.0584	1483 1538 1600 1624
				C	-0.3313	2.1335	0.3393	1634 1646 1656 2280
				C	-0.5505	3.3391	-0.1360	3074 3124 3146 3162
				H	-1.3232	4.0901	-0.0717	3166 3178 3181 3190
				H	-1.0917	-2.9222	0.0962	3195 3236 3243
				C	-1.6402	-0.4393	0.0190	
				C	-2.8140	-0.1446	-0.0576	
				C	-4.1548	0.2985	-0.1584	
				H	-4.2884	1.3653	-0.3277	
				C	-5.2365	-0.4893	-0.0650	
				H	-5.1505	-1.5565	0.1013	
				H	-6.2330	-0.0747	-0.1551	
				H	-1.0926	1.6981	0.9885	
CS27	0.211349	A	1696.79457	C	0.7034	1.7796	-0.1106	38 56 126 154
		B	4569.26463	C	0.4093	0.4237	-0.0924	180 216 226 319
		C	6164.12146	C	-0.9550	0.0123	-0.0170	346 357 441 474
				C	-1.9896	0.9973	0.0312	479 495 502 510
				C	-1.6371	2.3678	0.0062	535 563 575 610
				C	-0.3142	2.7463	-0.0621	646 680 702 751

				C	-1.3090	-1.3746	0.0123	759 775 797 807
				C	-3.3455	0.5724	0.1038	826 834 907 908
				H	-2.4232	3.1144	0.0430	915 927 981 988
				H	-0.0497	3.7977	-0.0787	992 1013 1055 1097
				C	-3.6620	-0.7642	0.1291	1118 1134 1152 1179
				C	-2.6438	-1.7387	0.0841	1187 1202 1222 1240
				H	-4.1265	1.3244	0.1393	1260 1298 1362 1382
				H	-4.6986	-1.0771	0.1848	1391 1419 1428 1465
				H	-2.9065	-2.7912	0.1068	1486 1492 1537 1611
				C	-0.2472	-2.3636	-0.0322	1621 1630 1668 1842
				C	1.0510	-2.0167	-0.1059	3074 3148 3158 3160
				H	1.8263	-2.7728	-0.1388	3161 3167 3173 3184
				H	1.7388	2.0971	-0.1624	3185 3188 3250
				C	1.4767	-0.6141	-0.1514	
				H	-0.5276	-3.4116	-0.0043	
				C	2.7624	-0.3167	-0.2475	
				C	4.0704	-0.0254	-0.3720	
				H	4.4583	0.0646	-1.3892	
				C	4.9713	0.1730	0.6770	
				H	4.6550	0.0989	1.7097	
				H	6.0080	0.4060	0.4727	
CS28	0.212432	A	1698.20085	C	0.0725	2.1273	-0.0473	50 77 136 165
		B	4422.81799	C	0.2597	0.7692	-0.0787	176 211 238 266
		C	6068.29012	C	-0.9480	-0.0070	-0.0252	348 389 446 457
				C	-2.2194	0.6424	0.0584	468 485 502 528
				C	-2.2839	2.0575	0.0852	537 595 613 646
				C	-1.1239	2.8149	0.0315	676 693 755 762
				C	-0.8858	-1.4344	-0.0560	773 787 807 827
				C	-3.3943	-0.1585	0.1151	837 891 907 912
				H	-3.2519	2.5442	0.1477	933 945 982 998
				H	-1.1607	3.8985	0.0503	1012 1031 1054 1095
				C	-3.3136	-1.5307	0.0868	1108 1132 1143 1183
				C	-2.0610	-2.1696	-0.0007	1202 1226 1257 1284
				H	-4.3584	0.3338	0.1821	1320 1332 1368 1376
				H	-4.2167	-2.1288	0.1305	1395 1429 1456 1462
				H	-2.0104	-3.2531	-0.0273	1480 1511 1572 1580
				C	0.4148	-2.0612	-0.1724	1614 1627 1650 1665
				C	1.5607	-1.3464	-0.2047	3109 3129 3143 3158
				H	2.5051	-1.8590	-0.3261	3160 3162 3169 3176
				C	1.5773	0.1030	-0.1315	3185 3208 3227
				H	0.4494	-3.1429	-0.2534	
				C	2.7232	0.8503	-0.1498	
				C	4.1115	0.4228	-0.1246	
				H	4.7936	1.0937	-0.6456	

				C	4.6549	-0.6291	0.5100	
				H	4.0731	-1.3027	1.1275	
				H	5.7214	-0.8140	0.4573	
				H	2.5875	1.9274	-0.2196	
CS29	0.215186	A	1790.74654	C	3.5684	-0.0370	-0.0004	86 98 183 205
		B	3350.31294	C	2.8914	1.1818	-0.0003	254 321 347 403
		C	5130.19078	C	1.4822	1.2266	-0.0002	431 452 486 493
				C	0.7499	-0.0031	-0.0001	505 513 541 541
				C	1.4525	-1.2455	-0.0001	590 655 660 683
				C	2.8692	-1.2355	-0.0003	732 736 749 786
				C	0.7580	2.4514	-0.0001	800 809 828 837
				C	-0.6775	0.0117	0.0001	884 936 943 952
				C	-1.3674	1.2703	0.0001	966 970 976 987
				C	-0.6237	2.4606	0.0000	998 1096 1134 1144
				C	-2.8135	1.2747	0.0003	1168 1191 1202 1209
				H	-3.3153	2.2377	0.0003	1215 1224 1242 1262
				C	-3.5363	0.1404	0.0004	1317 1338 1367 1380
				C	-2.9053	-1.2194	0.0003	1407 1431 1447 1448
				C	-1.3933	-1.2084	0.0002	1457 1493 1524 1545
				C	-0.6823	-2.4170	0.0001	1583 1594 1602 1660
				C	0.6993	-2.4468	0.0000	2960 2961 3147 3149
				H	1.2235	-3.3965	-0.0001	3155 3159 3168 3169
				H	-1.2348	-3.3521	0.0002	3173 3175 3183
				H	1.3095	3.3853	-0.0002	
				H	4.6528	-0.0472	-0.0005	
				H	3.4477	2.1130	-0.0004	
				H	3.4021	-2.1803	-0.0003	
				H	-1.1546	3.4073	0.0001	
				H	-4.6207	0.1850	0.0005	
				H	-3.2695	-1.7896	0.8693	
				H	-3.2697	-1.7896	-0.8685	
TS1	0.173804	A	834.13395	C	1.2285	0.8262	0.8253	-423 25 47 76
		B	3222.61892	C	1.7694	-0.3418	1.3747	139 153 309 389
		C	3499.69995	C	2.5906	-1.1016	0.5454	395 437 570 588
				C	2.9579	-0.6226	-0.7209	608 612 655 686
				C	2.5349	0.6401	-1.1554	712 724 780 832
				C	1.7127	1.4223	-0.3471	873 899 931 960
				C	-0.8143	0.1975	0.2029	968 980 983 990
				C	-1.1325	-1.0934	-0.1668	997 1014 1021 1049
				C	-2.4769	-1.3893	-0.4233	1076 1080 1168 1170
				C	-3.4490	-0.3951	-0.2987	1175 1180 1298 1307
				C	-3.0892	0.8985	0.0800	1315 1323 1429 1443
				C	-1.7462	1.2075	0.3323	1460 1474 1542 1562
				H	1.5136	-0.6746	2.3741	1576 1621 3144 3150

				H	2.9794	-2.0514	0.8988	3154 3160 3160 3165
				H	3.6194	-1.2115	-1.3457	3177 3178 3182 3188
				H	2.8799	1.0304	-2.1073	
				H	1.4204	2.4227	-0.6450	
				H	-0.3706	-1.8616	-0.2531	
				H	-2.7625	-2.3947	-0.7168	
				H	-4.4890	-0.6296	-0.4968	
				H	-3.8480	1.6687	0.1770	
				H	-1.4573	2.2125	0.6248	
TS2	0.178725	A	671.04825	C	-0.7268	-0.0434	-0.0114	-878 64 91 117
		B	3334.04079	C	-1.4522	-1.1958	-0.3725	182 263 281 314
		C	3837.48601	C	-2.8450	-1.2000	-0.3806	369 415 422 502
				C	-3.5625	-0.0566	-0.0300	558 615 620 635
				C	-2.8710	1.1032	0.3355	704 711 750 751
				C	-1.4932	1.0665	0.3246	785 856 860 913
				C	0.7581	-0.0324	-0.0073	933 953 966 985
				C	1.4822	-1.1354	0.4680	990 1002 1013 1020
				C	2.8745	-1.1306	0.4622	1026 1053 1070 1100
				C	3.5704	-0.0229	-0.0191	1130 1179 1182 1205
				C	2.8622	1.0804	-0.4930	1250 1296 1321 1322
				C	1.4701	1.0764	-0.4866	1355 1439 1476 1480
				H	-0.9104	-2.0864	-0.6730	1527 1575 1614 1628
				H	-3.3736	-2.1010	-0.6710	1641 2341 3158 3161
				H	-4.6470	-0.0640	-0.0374	3164 3169 3174 3179
				H	-3.4049	2.0016	0.6272	3186 3187 3193
				H	-0.8545	2.3017	0.8649	
				H	0.9510	-1.9924	0.8665	
				H	3.4161	-1.9898	0.8424	
				H	4.6545	-0.0189	-0.0235	
				H	3.3945	1.9446	-0.8745	
				H	0.9272	1.9312	-0.8719	
				H	-0.5538	3.0175	1.2125	
TSa	0.173588	A	1207.86033	C	0.7028	-0.3218	0.0547	-1184 51 57 83
		B	3358.15583	C	1.4273	-1.5169	0.2340	104 134 262 300
		C	4354.75361	C	2.8193	-1.5244	0.2370	359 409 418 424
				C	3.5407	-0.3432	0.0662	507 555 615 631
				C	2.8558	0.8639	-0.1131	639 697 710 737
				C	1.4788	0.8184	-0.1134	756 767 781 853
				C	-0.7794	-0.2891	0.0550	860 931 952 984
				C	-1.5215	-1.3160	-0.5453	991 1004 1011 1020
				C	-2.9129	-1.2803	-0.5464	1041 1044 1056 1088
				C	-3.5891	-0.2167	0.0507	1104 1141 1179 1183
				C	-2.8631	0.8107	0.6491	1206 1252 1298 1319
				C	-1.4710	0.7743	0.6555	1323 1356 1448 1475

				H	0.8823	-2.4406	0.3960	1484 1527 1575 1613
				H	3.3464	-2.4600	0.3853	1624 1638 3162 3164
				H	4.6248	-0.3525	0.0728	3167 3172 3176 3182
				H	3.3919	1.7952	-0.2543	3183 3191 3192
				H	0.8192	2.0064	-0.3835	
				H	-1.0065	-2.1330	-1.0377	
				H	-3.4699	-2.0783	-1.0245	
				H	-4.6727	-0.1886	0.0467	
				H	-3.3791	1.6405	1.1187	
				H	-0.9180	1.5634	1.1526	
				O	0.2819	2.9868	-0.5720	
TSb	0.185631	A	1298.63880	C	0.6474	-0.3613	0.0515	-1291 42 77 83
		B	3373.74805	C	1.2924	-1.5938	0.2749	114 131 153 264
		C	4490.24759	C	2.6795	-1.7026	0.2788	302 335 397 414
				C	3.4782	-0.5827	0.0571	446 504 559 619
				C	2.8714	0.6562	-0.1627	630 646 688 707
				C	1.4899	0.7312	-0.1605	742 759 784 791
				C	-0.8357	-0.2737	0.0375	852 863 927 949
				C	-1.6003	-1.3079	-0.5235	962 985 993 1003
				C	-2.9909	-1.2476	-0.5268	1014 1019 1036 1055
				C	-3.6458	-0.1508	0.0308	1081 1104 1130 1181
				C	-2.8973	0.8866	0.5840	1183 1210 1250 1289
				C	-1.5065	0.8281	0.5866	1313 1323 1360 1386
				H	0.6860	-2.4710	0.4710	1457 1482 1489 1530
				H	3.1387	-2.6672	0.4635	1582 1616 1624 1642
				H	4.5591	-0.6656	0.0545	3161 3163 3170 3172
				H	3.4729	1.5381	-0.3510	3179 3182 3189 3190
				H	1.0001	1.8522	-0.4169	3204 3767
				H	-1.1029	-2.1544	-0.9831	
				H	-3.5623	-2.0535	-0.9738	
				H	-4.7288	-0.1021	0.0280	
				H	-3.3966	1.7465	1.0162	
				H	-0.9382	1.6448	1.0124	
				O	0.7060	3.0757	-0.4596	
				H	1.3301	3.4359	0.1902	
TS3	0.205317	A	1800.44857	C	0.9390	-0.4193	-0.0655	-308 29 49 66
		B	3517.74237	C	1.5028	0.6223	-0.8174	83 108 133 193
		C	4975.47215	C	2.8788	0.8361	-0.8218	260 306 369 411
				C	3.7196	0.0132	-0.0736	416 430 498 558
				C	3.1722	-1.0250	0.6789	596 617 634 694
				C	1.7961	-1.2392	0.6829	704 713 746 750
				C	-0.5279	-0.6505	-0.0754	782 786 857 863
				C	-2.8237	0.2152	-0.0677	874 933 951 971
				C	-3.3246	-1.0893	-0.1437	988 989 1003 1013

				C	-2.4385	-2.1663	-0.1779	1024 1027 1049 1067
				C	-1.0626	-1.9528	-0.1446	1097 1123 1177 1181
				H	0.8535	1.2506	-1.4163	1201 1243 1295 1319
				H	3.2957	1.6409	-1.4172	1320 1353 1374 1433
				H	4.7909	0.1794	-0.0766	1470 1478 1524 1536
				H	3.8169	-1.6649	1.2711	1571 1612 1624 1639
				H	1.3791	-2.0360	1.2886	3045 3140 3155 3160
				H	-3.4948	1.0659	-0.0219	3161 3167 3172 3177
				H	-4.3959	-1.2589	-0.1719	3183 3185 3190 3240
				H	-2.8204	-3.1791	-0.2412	
				H	-0.3856	-2.7991	-0.2022	
				C	-1.4568	0.3824	-0.0363	
				C	-0.9146	2.4930	0.9107	
				H	0.0591	2.0742	1.1136	
				C	-1.4234	3.4751	0.1834	
				H	-2.4164	3.8788	0.3684	
				H	-0.8992	3.8794	-0.6862	
TS4	0.211355	A	1510.21073	C	-0.9427	-0.3219	0.0197	-863 47 76 86
		B	3626.94321	C	-1.6033	0.5731	0.8500	105 177 196 227
		C	4680.23304	C	-2.9689	0.7619	0.8793	257 289 317 329
				C	-3.7550	0.0068	0.0021	420 428 474 541
				C	-3.1437	-0.9075	-0.8551	567 581 620 626
				C	-1.7597	-1.0734	-0.8461	664 715 729 755
				C	0.5344	-0.5260	0.0427	777 794 811 864
				C	2.8224	0.2375	-0.1551	891 913 938 955
				C	3.2916	-1.0367	0.1353	967 969 994 997
				C	2.3867	-2.0704	0.3729	1012 1022 1028 1043
				C	1.0220	-1.8102	0.3199	1061 1080 1117 1134
				H	-0.8378	1.3214	1.8944	1178 1186 1220 1251
				H	-3.4224	1.4692	1.5657	1281 1306 1315 1324
				H	-4.8324	0.1316	-0.0063	1343 1439 1451 1469
				H	-3.7474	-1.4946	-1.5380	1484 1511 1574 1600
				H	-1.2941	-1.7801	-1.5254	1627 1635 1681 2367
				H	3.5311	1.0249	-0.3833	3137 3156 3157 3163
				H	4.3587	-1.2271	0.1602	3163 3172 3173 3182
				H	2.7404	-3.0706	0.5958	3186 3191 3220
				H	0.3136	-2.6070	0.5178	
				C	1.4482	0.5260	-0.2010	
				C	0.9780	1.8790	-0.5528	
				H	-0.0162	1.9339	-0.9854	
				C	1.6548	3.0183	-0.3804	
				H	2.6331	3.0520	0.0870	
				H	1.2338	3.9668	-0.6921	
				H	-0.4499	1.7410	2.5214	

TS5	0.199601	A	1309.57873	C	3.4963	-0.5814	-0.0754	-211 75 84 137
		B	3367.31637	C	2.8612	0.6243	-0.3427	152 244 272 317
		C	4535.13640	C	1.4708	0.7649	-0.2196	380 414 422 482
				C	0.6838	-0.3709	0.1045	503 561 585 602
				C	1.3517	-1.5733	0.3931	621 685 704 724
				C	2.7344	-1.6819	0.3160	742 773 781 813
				C	0.8617	2.0862	-0.4467	857 883 902 945
				C	-0.8051	-0.3512	0.0540	964 969 984 989
				C	-1.5754	0.7809	0.2782	993 1020 1041 1057
				C	0.0091	2.6566	0.4266	1072 1117 1143 1178
				C	-2.9491	0.8461	0.2260	1186 1221 1246 1269
				H	-3.4831	1.7712	0.4177	1291 1299 1316 1330
				C	-3.6445	-0.3286	-0.0839	1432 1438 1468 1484
				C	-2.9293	-1.4996	-0.3369	1506 1567 1591 1620
				C	-1.5385	-1.5133	-0.2754	1626 1636 3130 3142
				H	1.0811	2.5844	-1.3882	3152 3159 3162 3168
				H	4.5746	-0.6589	-0.1558	3171 3181 3185 3191
				H	3.4478	1.4922	-0.6255	3225
				H	0.7748	-2.4398	0.6928	
				H	3.2151	-2.6239	0.5549	
				H	-0.1002	2.2850	1.4375	
				H	-4.7284	-0.3239	-0.1350	
				H	-3.4584	-2.4094	-0.5974	
				H	-1.0118	-2.4310	-0.5120	
				H	-0.4741	3.6013	0.2027	
TS6	0.194996	A	1152.76480	C	-3.5689	-0.3039	-0.0214	-629 93 99 203
		B	3292.20982	C	-2.8418	0.8658	-0.0696	233 249 298 345
		C	4416.28897	C	-1.4272	0.8480	-0.0507	408 422 437 446
				C	-0.7395	-0.4001	0.0108	506 513 548 557
				C	-1.5144	-1.5786	0.0634	600 631 722 725
				C	-2.8958	-1.5359	0.0486	730 751 767 797
				C	-0.6842	2.0664	-0.0951	840 842 873 880
				C	0.7193	-0.4087	0.0034	887 957 966 987
				C	1.4219	0.8269	-0.0206	992 998 1016 1060
				C	0.6847	2.0682	-0.0175	1061 1113 1164 1167
				C	2.8319	0.8308	-0.0437	1182 1188 1222 1237
				H	3.3503	1.7838	-0.0563	1264 1304 1321 1364
				C	3.5464	-0.3492	-0.0448	1372 1440 1448 1463
				C	2.8597	-1.5744	-0.0203	1488 1528 1556 1605
				C	1.4788	-1.6001	0.0028	1618 1643 1651 3160
				H	-1.2273	3.0035	-0.1526	3161 3164 3170 3171
				H	-4.6525	-0.2761	-0.0337	3181 3184 3187 3194
				H	-3.3485	1.8240	-0.1192	3207
				H	-1.0276	-2.5435	0.1194	

				H	-3.4621	-2.4594	0.0915	
				H	1.2364	2.9919	-0.1486	
				H	4.6301	-0.3329	-0.0638	
				H	3.4147	-2.5057	-0.0213	
				H	0.9789	-2.5598	0.0155	
				H	0.8822	2.5434	1.8973	
TS7	0.194677	A	1062.12556	C	0.7115	0.1450	-0.7335	-199 -5 21 43
		B	6202.77873	C	-0.1484	-0.8868	-0.5383	65 94 175 199
		C	6576.38449	C	-1.4914	-0.5821	-0.1570	232 328 369 388
				C	-1.8669	0.7910	0.0089	473 482 494 513
				C	-0.9000	1.8099	-0.2091	561 615 623 650
				C	0.3925	1.4972	-0.5782	673 694 744 758
				H	-2.1772	-2.6306	-0.0610	766 790 807 848
				H	0.1507	-1.9234	-0.6604	883 897 931 939
				C	-2.4638	-1.5917	0.0649	957 970 991 996
				C	-3.2016	1.0913	0.3902	1037 1046 1110 1142
				H	-1.1937	2.8473	-0.0788	1160 1172 1208 1250
				C	-4.1204	0.0905	0.5979	1276 1314 1339 1384
				C	-3.7472	-1.2642	0.4335	1392 1440 1456 1464
				H	-3.4867	2.1308	0.5160	1527 1590 1613 1636
				H	-5.1356	0.3357	0.8887	1652 2111 3135 3147
				H	-4.4802	-2.0459	0.6001	3150 3153 3157 3162
				C	3.0535	-0.4234	-1.4291	3173 3174 3186 3238
				H	2.6695	-0.3956	-2.4200	3456
				C	3.7504	-0.5120	-0.4374	
				C	4.4702	-0.6333	0.7735	
				H	4.4945	-1.6274	1.2136	
				C	5.1078	0.3758	1.3880	
				H	5.1078	1.3798	0.9810	
				H	5.6428	0.2097	2.3149	
				H	1.1302	2.2754	-0.7404	
TS8	0.193965	A	1098.41344	C	-0.8731	1.0534	-0.1298	-1952 56 72 129
		B	4830.22595	C	-0.3072	-0.2014	-0.1587	145 170 218 286
		C	5841.76477	C	1.0601	-0.4593	-0.0595	310 352 422 460
				C	1.9156	0.7005	0.0524	491 500 519 533
				C	1.3348	1.9951	0.0690	592 646 661 678
				C	-0.0299	2.1811	-0.0177	753 757 760 789
				H	1.0007	-2.6199	-0.1542	819 865 876 929
				H	-1.5640	-0.8949	-0.2749	935 958 971 973
				C	1.6490	-1.7551	-0.0702	990 1000 1018 1035
				C	3.3185	0.4964	0.1435	1038 1095 1140 1162
				H	1.9925	2.8538	0.1556	1174 1185 1232 1258
				C	3.8530	-0.7688	0.1316	1305 1313 1367 1371
				C	3.0097	-1.9037	0.0253	1380 1436 1458 1481

				H	3.9662	1.3636	0.2249	1537 1582 1606 1618
				H	4.9261	-0.9054	0.2034	1651 1670 1728 3113
				H	3.4482	-2.8953	0.0176	3137 3155 3157 3159
				C	-2.3452	0.9752	-0.2129	3166 3174 3179 3189
				H	-2.9894	1.8487	-0.2484	3227
				C	-2.7780	-0.2876	-0.2706	
				C	-4.0270	-0.9866	-0.2806	
				H	-4.1633	-1.7292	-1.0658	
				C	-5.0041	-0.8214	0.6304	
				H	-4.8956	-0.1266	1.4555	
				H	-5.9290	-1.3832	0.5672	
				H	-0.4500	3.1815	0.0072	
TS9	0.195035	A	1196.09194	C	-1.0597	0.8814	-0.2986	-487 57 66 94
		B	4531.10693	C	-0.3221	-0.2985	-0.3987	142 186 240 266
		C	5319.35802	C	1.0483	-0.4598	-0.2082	327 389 415 431
				C	1.7860	0.7313	0.1327	496 508 513 524
				C	1.0732	1.9704	0.2453	578 619 624 653
				C	-0.2710	2.0532	0.0425	722 748 755 761
				H	1.1820	-2.5848	-0.5822	769 817 818 869
				H	-3.4217	-0.4090	-1.9402	919 956 960 971
				C	1.7426	-1.6936	-0.3259	988 988 993 1038
				C	3.1763	0.6281	0.3396	1041 1131 1159 1162
				H	1.6359	2.8626	0.4998	1169 1189 1228 1236
				C	3.8255	-0.5858	0.2189	1266 1327 1344 1387
				C	3.1024	-1.7506	-0.1159	1404 1427 1433 1444
				H	3.7352	1.5224	0.5968	1454 1509 1520 1556
				H	4.8955	-0.6462	0.3815	1607 1629 3100 3107
				H	3.6237	-2.6968	-0.2075	3136 3145 3153 3157
				C	-2.4417	0.9427	-0.5159	3169 3172 3182 3192
				H	-2.9299	1.9147	-0.4556	3238
				C	-3.2541	-0.2274	-0.8789	
				C	-3.8841	-1.0785	0.0307	
				H	-4.4718	-1.8892	-0.3942	
				C	-3.8303	-0.9787	1.4054	
				H	-3.2582	-0.1987	1.8936	
				H	-4.3549	-1.6849	2.0363	
				H	-0.7831	3.0060	0.1330	
TS11	0.195009	A	1145.00867	C	1.3959	0.8992	0.0132	-627 96 99 223
		B	3292.65329	C	0.7127	-0.3470	-0.0187	224 245 283 351
		C	4410.69391	C	-0.7386	-0.3623	-0.0187	408 413 447 457
				C	-1.4446	0.8759	-0.0057	504 508 553 556
				C	-0.7135	2.1085	0.0221	599 630 719 724
				C	0.6440	2.1200	0.0359	726 747 774 798
				H	-0.9932	-2.5150	-0.0153	832 842 873 885

				H	4.6296	-0.2045	-0.0922	893 960 973 983
				C	-1.4955	-1.5566	-0.0224	992 999 1014 1059
				C	-2.8577	0.8734	-0.0128	1061 1109 1160 1172
				H	-1.2717	3.0388	0.0356	1184 1187 1221 1240
				C	-3.5669	-0.3087	-0.0247	1261 1300 1327 1364
				C	-2.8759	-1.5332	-0.0260	1372 1440 1444 1465
				H	-3.3797	1.8247	-0.0057	1485 1530 1554 1597
				H	-4.6509	-0.2966	-0.0288	1625 1646 1655 3156
				H	-3.4292	-2.4656	-0.0275	3160 3162 3170 3174
				C	2.8101	0.9242	0.0022	3176 3185 3188 3196
				H	3.3126	1.8854	0.0292	3208
				C	3.5467	-0.2432	-0.0623	
				C	2.8877	-1.4768	-0.1008	
				H	3.4572	-2.3970	-0.1600	
				C	1.4964	-1.5364	-0.0288	
				H	1.3190	-2.0577	1.8649	
				H	1.0157	-2.4940	-0.1753	
				H	1.1866	3.0592	0.0603	
TS14	0.224210	A	2166.16631	C	-3.6370	0.7904	-0.0369	-1049 63 99 123
		B	3586.53199	C	-3.2261	-0.5129	-0.2239	152 186 225 256
		C	5647.20827	C	-1.8629	-0.8758	-0.1454	262 297 349 395
				C	-0.8600	0.1296	0.0579	410 421 480 493
				C	-1.3592	1.4136	0.3320	513 514 540 576
				C	-2.6824	1.7695	0.2985	581 612 672 690
				C	-1.4766	-2.2545	-0.1952	721 736 763 777
				C	0.5488	-0.2479	0.0242	799 816 840 846
				C	0.8608	-1.6401	0.0913	895 920 935 956
				C	-0.1832	-2.6185	-0.0171	973 977 981 987
				C	2.2004	-2.0639	0.2059	1010 1028 1049 1056
				H	2.4083	-3.1260	0.2792	1107 1116 1143 1173
				C	3.2286	-1.1460	0.1900	1191 1196 1226 1250
				C	2.9391	0.2069	-0.0104	1275 1295 1329 1341
				C	1.6311	0.6805	-0.1108	1352 1406 1422 1446
				H	-2.2500	-3.0031	-0.3313	1450 1468 1518 1522
				H	-4.6878	1.0520	-0.0949	1584 1622 1637 1651
				H	-3.9569	-1.2931	-0.4083	1677 2122 3136 3147
				H	-0.6241	2.4061	1.0622	3157 3159 3163 3167
				H	-2.9903	2.7773	0.5568	3176 3179 3182 3193
				H	0.0958	-3.6666	0.0065	3219
				H	4.2594	-1.4722	0.2694	
				H	3.7552	0.9049	-0.1517	
				C	1.4260	2.0884	-0.5036	
				H	0.5485	2.2809	-1.1140	
				C	2.2293	3.1144	-0.2095	

				H	3.0932	3.0105	0.4382	
				H	2.0273	4.1065	-0.5954	
				H	-0.3368	3.0201	1.6014	
TS15	0.212331	A	1970.83548	C	-3.5029	-0.3570	-0.0993	-238 71 107 154
		B	3378.37695	C	-2.6954	-1.4641	0.0481	204 221 251 300
		C	5276.43851	C	-1.2901	-1.3336	0.0830	386 405 432 455
				C	-0.6850	-0.0434	0.0143	486 499 507 554
				C	-1.5431	1.0958	-0.1065	563 571 622 636
				C	-2.9205	0.9122	-0.1938	691 721 732 758
				C	-0.4716	-2.5106	0.1467	772 801 808 839
				C	0.7731	0.0316	-0.0206	841 891 919 939
				C	1.5477	-1.1737	-0.0027	968 971 974 980
				C	0.8816	-2.4378	0.0962	986 1006 1061 1074
				C	2.9592	-1.1029	-0.0680	1110 1148 1172 1190
				H	3.5231	-2.0296	-0.0533	1193 1224 1254 1256
				C	3.6189	0.1060	-0.1447	1297 1305 1342 1350
				C	2.8748	1.3080	-0.1454	1403 1422 1439 1455
				C	1.5145	1.2146	-0.0854	1468 1507 1522 1581
				C	-0.0461	2.9625	0.5906	1614 1631 1642 1651
				C	-1.0226	2.4687	-0.1952	3133 3145 3151 3155
				H	-1.4281	3.0894	-0.9905	3160 3161 3167 3174
				H	0.3416	3.9629	0.4335	3177 3186 3227
				H	-0.9676	-3.4730	0.2156	
				H	-4.5807	-0.4636	-0.1464	
				H	-3.1282	-2.4566	0.1139	
				H	-3.5541	1.7845	-0.3156	
				H	1.4847	-3.3395	0.1281	
				H	4.7023	0.1388	-0.1953	
				H	3.3808	2.2671	-0.1899	
				H	0.2703	2.4613	1.4959	
TS16	0.207579	A	1827.13716	C	3.5292	0.0089	-0.0212	-629 96 147 196
		B	3264.93038	C	2.8286	1.2107	-0.0524	237 251 299 346
		C	5063.68577	C	1.4245	1.2234	-0.0417	358 410 420 461
				C	0.7204	-0.0189	-0.0027	498 506 508 516
				C	1.4470	-1.2446	0.0230	550 554 582 594
				C	2.8502	-1.2055	0.0165	685 703 723 748
				C	0.6719	2.4386	-0.0779	762 772 813 814
				C	-0.7063	-0.0336	-0.0059	826 832 862 906
				C	-1.4363	1.1892	-0.0290	919 977 979 984
				C	-0.6998	2.4335	-0.0133	988 997 1014 1090
				C	-2.8348	1.1497	-0.0553	1113 1124 1157 1166
				H	-3.3916	2.0806	-0.0685	1197 1203 1227 1258
				C	-3.5119	-0.0679	-0.0545	1262 1265 1342 1349
				C	-2.8091	-1.2677	-0.0225	1396 1418 1431 1456

				C	-1.4062	-1.2764	0.0016	1458 1478 1503 1531
				C	-0.6456	-2.4944	0.0332	1581 1619 1625 1632
				C	0.7134	-2.4795	0.0462	1659 3157 3160 3160
				H	1.2713	-3.4098	0.0694	3164 3168 3169 3176
				H	-1.1829	-3.4370	0.0454	3181 3185 3185
				H	1.2084	3.3804	-0.1207	
				H	4.6134	0.0185	-0.0268	
				H	3.3655	2.1529	-0.0831	
				H	3.4049	-2.1376	0.0379	
				H	-0.9088	2.8926	1.9121	
				H	-4.5958	-0.0796	-0.0748	
				H	-3.3443	-2.2114	-0.0167	
				H	-1.2519	3.3577	-0.1391	
TS17	0.203719	A	2087.36762	C	0.9892	-0.4593	-0.7443	-192 9 20 42
		B	6530.46861	C	0.0224	0.4730	-0.5434	57 92 110 142
		C	7840.18963	C	-1.2715	0.0243	-0.1438	177 210 231 329
				C	-1.4881	-1.3807	0.0324	357 362 437 441
				C	-0.4164	-2.2873	-0.1940	480 490 530 562
				C	0.8286	-1.8367	-0.5801	571 588 631 644
				H	0.2003	1.5347	-0.6730	650 675 694 696
				C	-2.3628	0.9273	0.0905	700 747 774 787
				C	-2.7697	-1.8399	0.4316	827 860 873 898
				H	-0.5947	-3.3495	-0.0559	926 942 961 985
				H	1.6469	-2.5275	-0.7495	997 1024 1054 1093
				C	-3.7995	-0.9557	0.6499	1111 1151 1190 1218
				C	-3.5968	0.4273	0.4796	1241 1278 1315 1327
				H	-2.9249	-2.9057	0.5625	1359 1386 1440 1446
				H	-4.7750	-1.3166	0.9546	1469 1513 1593 1609
				H	-4.4127	1.1185	0.6532	1635 1637 2114 2194
				C	-2.1888	2.3336	-0.0740	3135 3148 3153 3163
				C	-2.0482	3.5231	-0.2117	3172 3176 3180 3194
				H	-1.9289	4.5721	-0.3312	3239 3456 3475
				C	3.2653	0.3611	-1.4292	
				H	2.7956	0.6170	-2.3480	
				C	4.0360	0.1942	-0.5049	
				C	4.8687	-0.0684	0.6074	
				H	5.4289	-0.9999	0.5738	
				C	5.0046	0.7458	1.6661	
				H	4.4629	1.6814	1.7358	
				H	5.6637	0.4843	2.4847	
TS18	0.202777	A	2058.59861	C	-0.9494	-1.4321	-0.1456	-1956 44 59 99
		B	4855.54177	C	-0.3547	-0.1888	-0.1343	112 146 156 179
		C	6824.05807	C	1.0160	0.0318	-0.0287	221 285 304 339
				C	1.8444	-1.1490	0.0449	387 437 452 470

				C	1.2334	-2.4297	0.0217	506 525 534 588
				C	-0.1348	-2.5822	-0.0695	588 594 632 669
				H	-1.5898	0.5381	-0.2286	681 691 736 745
				C	1.6397	1.3270	0.0003	757 781 831 874
				C	3.2513	-0.9913	0.1401	884 925 930 961
				H	1.8725	-3.3043	0.0803	963 981 999 1003
				H	-0.5777	-3.5729	-0.0762	1038 1055 1088 1098
				C	3.8217	0.2588	0.1668	1159 1182 1190 1238
				C	3.0176	1.4166	0.0983	1253 1292 1311 1346
				H	3.8729	-1.8792	0.1934	1364 1394 1435 1451
				H	4.8978	0.3654	0.2408	1463 1532 1582 1606
				H	3.4805	2.3957	0.1211	1614 1635 1668 1731
				C	0.8428	2.5066	-0.0693	2196 3115 3137 3157
				C	0.1777	3.5103	-0.1278	3160 3161 3176 3179
				H	-0.4089	4.3947	-0.1772	3194 3227 3476
				C	-2.4185	-1.3148	-0.2284	
				H	-3.0847	-2.1698	-0.2924	
				C	-2.8153	-0.0388	-0.2466	
				C	-4.0448	0.6937	-0.2393	
				H	-4.1544	1.4667	-0.9987	
				C	-5.0309	0.5240	0.6609	
				H	-4.9458	-0.2011	1.4623	
				H	-5.9391	1.1138	0.6139	
TS19	0.208177	A	2192.56852	C	-1.4364	-1.1783	-0.1892	-275 36 82 127
		B	3756.43505	C	-0.6267	-0.0659	-0.1205	137 178 197 208
		C	5870.12516	C	0.7756	-0.0840	-0.0352	289 327 370 402
				C	1.4016	-1.3801	0.0341	407 450 478 489
				C	0.5845	-2.5419	-0.0338	513 542 575 589
				C	-0.7745	-2.4502	-0.1485	611 633 675 681
				C	1.6209	1.0787	-0.0157	697 708 752 765
				C	2.8079	-1.4742	0.1572	798 802 834 843
				H	1.0639	-3.5142	0.0036	911 925 933 964
				H	-1.3777	-3.3512	-0.2046	965 982 984 996
				C	3.5897	-0.3431	0.1968	1039 1082 1095 1171
				C	2.9966	0.9279	0.1033	1173 1195 1225 1243
				H	3.2605	-2.4585	0.2200	1259 1286 1316 1352
				H	4.6663	-0.4236	0.2925	1380 1423 1445 1458
				H	3.6174	1.8154	0.1182	1474 1509 1552 1582
				C	1.1003	2.4007	-0.1371	1612 1622 1649 2193
				C	0.7458	3.5471	-0.2526	3123 3134 3151 3155
				H	0.4204	4.5532	-0.3565	3158 3161 3175 3180
				C	-2.8967	-1.1755	-0.2268	3195 3248 3474
				H	-3.3487	-2.1396	-0.4427	
				C	-3.7233	-0.1677	0.1516	

				C	-3.2644	1.1450	0.5287	
				H	-3.7245	1.6058	1.4007	
				C	-2.2375	1.7769	-0.0956	
				H	-1.9513	1.5196	-1.1062	
				H	-1.8291	2.6998	0.2932	
				H	-4.7775	-0.3995	0.2826	
TS20	0.206248	A	2131.69932	C	-3.5412	0.4710	-0.1917	-1991 70 121 159
		B	3307.37061	C	-3.0376	-0.8276	-0.2818	204 225 264 303
		C	5353.83143	C	-1.6616	-1.0862	-0.0607	338 378 415 444
				C	-0.8023	-0.0102	0.2470	492 500 512 521
				C	-1.3474	1.3163	0.5087	551 561 580 596
				C	-2.7402	1.5198	0.2384	625 651 699 712
				C	-1.1002	-2.4039	-0.1507	730 738 771 788
				C	0.6120	-0.1845	0.1747	808 833 858 863
				C	1.1478	-1.5056	0.0999	923 965 972 977
				C	0.2393	-2.6083	-0.0154	985 988 1007 1047
				C	2.5439	-1.6815	0.0640	1095 1120 1157 1171
				H	2.9490	-2.6877	0.0434	1190 1212 1227 1248
				C	3.3886	-0.5858	0.0147	1264 1310 1337 1340
				C	2.8642	0.7105	-0.0511	1362 1402 1415 1439
				C	1.4904	0.9341	0.0266	1452 1479 1516 1531
				H	-1.7685	-3.2412	-0.3231	1562 1586 1617 1638
				H	-4.5927	0.6489	-0.3903	1850 3045 3158 3160
				H	-3.6961	-1.6514	-0.5342	3163 3173 3177 3179
				H	-0.9780	1.8194	1.4057	3184 3192 3371
				H	-3.1801	2.4911	0.4356	
				H	0.6484	-3.6123	-0.0575	
				H	4.4629	-0.7286	-0.0150	
				H	3.5232	1.5606	-0.1801	
				H	-0.4149	2.0653	-0.2001	
				C	0.9588	2.2742	-0.2066	
				C	1.3113	3.4739	-0.3527	
				H	1.0929	4.4992	-0.5639	
TS21	0.211518	A	1906.44019	C	-3.5651	0.0378	0.0260	-352 93 132 163
		B	3375.99994	C	-2.8776	-1.1272	-0.2706	221 226 278 300
		C	5123.53423	C	-1.4672	-1.1837	-0.1645	389 414 434 477
				C	-0.7443	-0.0089	0.1703	495 504 513 550
				C	-1.4773	1.1457	0.5885	574 594 603 658
				C	-2.8751	1.1533	0.5113	673 720 727 757
				C	-0.7548	-2.4194	-0.3103	772 779 810 831
				C	0.6972	-0.0232	0.1200	838 870 876 890
				C	1.3611	-1.2837	0.0841	917 920 972 977
				C	0.5893	-2.4761	-0.1159	983 1008 1038 1062
				C	2.7693	-1.3381	0.1736	1105 1139 1168 1175

				H	3.2579	-2.3066	0.1851	1189 1222 1223 1246
				C	3.5133	-0.1772	0.2110	1258 1284 1331 1346
				C	2.8717	1.0613	0.0703	1365 1415 1438 1458
				C	1.4852	1.1658	-0.0065	1465 1518 1535 1581
				H	-1.3186	-3.3196	-0.5313	1605 1619 1640 1645
				H	-4.6466	0.0627	-0.0507	3038 3157 3159 3160
				H	-3.4169	-2.0227	-0.5605	3166 3170 3176 3180
				H	-0.9982	1.8446	1.2601	3185 3188 3219
				H	-3.4243	2.0175	0.8677	
				H	1.1124	-3.4255	-0.1608	
				H	4.5942	-0.2200	0.2819	
				H	3.4695	1.9612	-0.0319	
				C	0.9212	2.4605	-0.4389	
				C	-0.3538	2.7728	-0.5792	
				H	-0.8641	3.6627	-0.9208	
				H	1.6763	3.1875	-0.7543	
TS22	0.208065	A	1807.55685	C	-3.5332	-0.0240	-0.0231	-1015 95 148 208
		B	3269.55111	C	-2.8595	1.1913	-0.0016	236 262 350 395
		C	5048.17056	C	-1.4559	1.2335	0.0128	403 441 459 493
				C	-0.7266	0.0070	0.0164	503 505 519 534
				C	-1.4279	-1.2362	-0.0214	545 561 584 596
				C	-2.8267	-1.2268	-0.0389	688 698 721 747
				C	-0.7229	2.4670	0.0173	750 774 809 812
				C	0.6945	0.0271	0.0298	828 838 859 899
				C	1.3961	1.2626	0.0026	914 975 976 982
				C	0.6371	2.4814	0.0043	984 990 1010 1089
				C	2.8012	1.2517	-0.0481	1112 1122 1164 1167
				H	3.3360	2.1950	-0.0740	1194 1202 1221 1250
				C	3.5062	0.0488	-0.1109	1261 1265 1334 1345
				C	2.8426	-1.1659	-0.0611	1389 1425 1430 1447
				C	1.4315	-1.2119	0.1195	1455 1472 1511 1524
				C	0.6864	-2.4533	-0.0209	1583 1609 1621 1640
				C	-0.6654	-2.4586	-0.0723	1665 3157 3159 3161
				H	-1.2048	-3.3960	-0.1591	3162 3168 3172 3176
				H	1.2464	-3.3816	-0.0542	3179 3185 3186
				H	-1.2800	3.3981	0.0215	
				H	-4.6172	-0.0383	-0.0347	
				H	-3.4165	2.1223	0.0005	
				H	-3.3626	-2.1697	-0.0690	
				H	1.1748	3.4238	-0.0060	
				H	4.5863	0.0663	-0.2044	
				H	3.3953	-2.0979	-0.1008	
				H	1.4770	-1.2840	1.8538	
TS23	0.204130	A	2113.11016	C	-0.9851	-1.4892	-0.1654	-273 11 27 51

		B	5190.55442	C	-0.3695	-0.2771	-0.1114	98 119 144 164
		C	7202.28323	C	1.0258	-0.0929	-0.0266	174 203 233 341
				C	1.8187	-1.2991	0.0073	360 368 439 458
				C	1.1761	-2.5638	-0.0492	483 485 543 559
				C	-0.1898	-2.6630	-0.1351	577 591 627 639
				C	1.7138	1.1680	0.0262	641 685 704 710
				C	3.2309	-1.2062	0.0965	745 753 760 799
				H	1.7916	-3.4566	-0.0230	816 865 900 902
				H	-0.6698	-3.6350	-0.1777	927 933 976 987
				C	3.8582	0.0141	0.1492	996 1019 1064 1098
				C	3.0991	1.1973	0.1130	1111 1163 1188 1202
				H	3.8092	-2.1240	0.1221	1236 1260 1310 1345
				H	4.9381	0.0747	0.2171	1355 1383 1437 1453
				H	3.5967	2.1585	0.1519	1465 1511 1582 1616
				C	1.0331	2.4220	-0.0107	1623 1645 2071 2190
				C	0.5466	3.5253	-0.0384	3130 3146 3158 3162
				H	0.1089	4.4934	-0.0660	3174 3180 3184 3196
				H	-2.0640	-1.5685	-0.2264	3238 3408 3471
				C	-2.1646	1.3590	-0.1198	
				H	-1.4171	2.1128	-0.0335	
				C	-3.2500	0.8087	-0.2061	
				C	-4.4421	0.0687	-0.3448	
				H	-4.7870	-0.0951	-1.3637	
				C	-5.1634	-0.4225	0.6802	
				H	-4.8575	-0.2773	1.7094	
				H	-6.0754	-0.9782	0.4997	
TS24	0.202104	A	2099.36290	C	-0.2826	-2.2159	-0.1023	-1725 36 57 92
		B	4585.77019	C	-0.3114	-0.8300	-0.0996	121 154 183 222
		C	6580.90465	C	0.9164	-0.0931	-0.0213	235 304 321 340
				C	2.1483	-0.8200	0.0518	374 396 474 494
				C	2.1239	-2.2381	0.0457	509 533 548 552
				C	0.9331	-2.9199	-0.0297	577 590 638 647
				C	0.9628	1.3390	-0.0140	660 719 755 772
				C	3.3733	-0.1095	0.1292	776 804 828 853
				H	3.0644	-2.7756	0.1022	877 885 908 918
				H	0.9222	-4.0040	-0.0337	935 967 983 991
				C	3.3890	1.2650	0.1347	1019 1048 1103 1107
				C	2.1847	1.9877	0.0630	1155 1189 1202 1239
				H	4.2991	-0.6720	0.1845	1253 1288 1296 1365
				H	4.3296	1.8008	0.1944	1377 1388 1429 1457
				H	2.1980	3.0710	0.0672	1481 1499 1533 1568
				C	-0.2764	2.1018	-0.0887	1607 1623 1644 1832
				C	-0.6932	3.2985	-0.1140	2032 3113 3147 3162
				H	-1.5334	3.9618	-0.1651	3165 3178 3179 3191

				H	-1.2193	-2.7573	-0.1618	3192 3242 3344
				C	-1.5808	-0.1293	-0.1769	
				H	-1.2125	1.2030	-0.1481	
				C	-2.8264	-0.2614	-0.2497	
				C	-4.2088	-0.1702	-0.3599	
				H	-4.6089	-0.1605	-1.3731	
				C	-5.0786	-0.1172	0.6838	
				H	-4.7330	-0.1306	1.7103	
				H	-6.1457	-0.0605	0.5104	
TS26	0.206637	A	1678.59816	C	0.5185	1.8837	-0.1282	-2060 53 78 126
		B	4361.71709	C	0.3110	0.5261	-0.1171	164 180 203 252
		C	5959.88990	C	-0.9749	-0.0191	-0.0298	320 360 395 450
				C	-2.0846	0.8670	0.0722	458 481 497 507
				C	-1.8333	2.2639	0.0904	531 552 596 627
				C	-0.5379	2.7806	0.0040	632 684 693 746
				C	-1.1239	-1.4384	-0.0470	758 771 801 805
				C	-3.3798	0.2775	0.1559	835 836 907 911
				H	-2.6743	2.9464	0.1636	920 951 981 990
				H	-0.3835	3.8540	0.0189	998 1024 1046 1062
				C	-3.5238	-1.0919	0.1378	1085 1128 1144 1169
				C	-2.4059	-1.9565	0.0353	1179 1203 1237 1253
				H	-4.2524	0.9172	0.2349	1309 1315 1350 1381
				H	-4.5158	-1.5252	0.2016	1418 1435 1440 1451
				H	-2.5611	-3.0302	0.0169	1469 1518 1573 1581
				C	0.0862	-2.2468	-0.1658	1596 1597 1632 1652
				C	1.3380	-1.7203	-0.2361	1711 3110 3137 3156
				H	2.2000	-2.3711	-0.3246	3157 3160 3168 3175
				H	1.9439	1.7316	-0.2497	3183 3186 3227
				C	1.5244	-0.2831	-0.1885	
				H	-0.0378	-3.3250	-0.1971	
				C	2.6242	0.5106	-0.2277	
				C	4.0398	0.3798	-0.1570	
				H	4.6225	1.0634	-0.7736	
				C	4.7038	-0.4925	0.6320	
				H	4.1808	-1.1611	1.3063	
				H	5.7864	-0.5438	0.6193	
TS27	0.211684	A	1802.24157	C	-1.1395	1.3775	0.0608	-148 54 118 169
		B	3736.80475	C	-0.6211	0.1029	0.0480	197 216 242 296
		C	5497.45166	C	0.8166	0.0182	-0.0029	377 394 434 460
				C	1.6029	1.2119	-0.0490	471 480 503 533
				C	0.9600	2.4770	-0.0430	543 585 616 642
				C	-0.4180	2.5626	0.0245	656 680 757 764
				C	1.4759	-1.2487	0.0341	772 774 798 822
				C	3.0184	1.1037	-0.0862	833 877 891 909

				H	1.5659	3.3771	-0.0820	953 962 973 980
				H	-0.9147	3.5272	0.0389	987 1010 1050 1094
				C	3.6342	-0.1279	-0.0597	1117 1140 1152 1182
				C	2.8653	-1.3020	0.0063	1201 1233 1240 1262
				H	3.6095	2.0122	-0.1305	1309 1320 1362 1376
				H	4.7159	-0.1956	-0.0852	1412 1432 1445 1457
				H	3.3580	-2.2683	0.0356	1480 1500 1527 1579
				C	0.6640	-2.4413	0.1080	1607 1614 1627 1654
				C	-0.6859	-2.3804	0.1115	3109 3130 3145 3152
				H	-1.2657	-3.2966	0.1489	3156 3160 3167 3169
				C	-1.4219	-1.1325	0.0451	3176 3185 3234
				H	1.1671	-3.4016	0.1521	
				C	-2.7842	-1.2023	-0.1551	
				C	-3.7317	-0.1363	-0.3243	
				H	-4.5329	-0.3364	-1.0349	
				C	-3.7279	1.0659	0.2966	
				H	-4.4522	1.8280	0.0299	
				H	-3.1147	1.2653	1.1636	
				H	-3.1778	-2.2021	-0.3246	
TS28	0.207480	A	1804.91214	C	-3.5498	-0.0598	0.0199	-560 96 144 207
		B	3299.13279	C	-2.8851	1.1633	0.0312	225 254 293 316
		C	5075.30141	C	-1.4832	1.2178	0.0182	363 410 412 462
				C	-0.7432	-0.0015	-0.0047	497 506 508 509
				C	-1.4320	-1.2502	-0.0178	544 553 585 595
				C	-2.8354	-1.2540	-0.0053	688 701 720 747
				C	-0.7615	2.4586	0.0243	760 780 811 814
				C	0.6818	0.0276	-0.0151	821 833 856 910
				C	1.3698	1.2780	-0.0195	934 977 979 982
				C	0.5990	2.4864	0.0039	986 993 1011 1084
				C	2.7763	1.2841	-0.0604	1109 1128 1166 1167
				H	3.2989	2.2345	-0.0843	1197 1204 1226 1254
				C	3.4924	0.0988	-0.0773	1260 1267 1343 1349
				C	2.8306	-1.1380	-0.0228	1398 1421 1434 1453
				C	1.4187	-1.1909	-0.0381	1457 1484 1504 1528
				C	0.7010	-2.4302	-0.0550	1582 1618 1622 1635
				C	-0.6595	-2.4595	-0.0457	1657 3157 3158 3161
				H	-1.1862	-3.4080	-0.0574	3164 3168 3174 3176
				H	1.2679	-3.3551	-0.0686	3177 3185 3189
				H	-1.3273	3.3843	0.0428	
				H	-4.6337	-0.0823	0.0299	
				H	-3.4505	2.0890	0.0492	
				H	-3.3621	-2.2023	-0.0156	
				H	1.1265	3.4346	0.0047	
				H	4.5756	0.1214	-0.1097	

H	3.3926	-2.0570	-0.1404
H	3.1786	-1.5415	1.9357
